# System-wide assembly of pathways and modules hierarchically reveal metabolic mechanism of cerebral ischemia

**DOI:** 10.1038/srep17068

**Published:** 2015-12-01

**Authors:** Yan Zhu, Zhili Guo, Liangxiao Zhang, Yingying Zhang, Yinying Chen, Jingyi Nan, Buchang Zhao, Hongbin Xiao, Zhong Wang, Yongyan Wang

**Affiliations:** 1 Institute of Basic Research in Clinical Medicine, China Academy of Chinese Medical Sciences, Beijing, 100700, China; 2 Beijing Electric Power Hospital, Capital Medical University, Beijing, 100073, China; 3 Beijing University of Chinese Medicine, Beijing, 100029, China; 4 Shanxi Buchang Pharmaceutical Co. Ltd, Xi’an, 712000, China; 5 Jiaxing Traditional Chinese Medicine Affiliated Hospital of Zhejiang Chinese Medical University, Jiaxing, 314000, China; 6 Oil Crops Research Institute, Chinese Academy of Agricultural Sciences, Wuhan 430062, China

## Abstract

The relationship between cerebral ischemia and metabolic disorders is poorly understood, which is partly due to the lack of comparative fusing data for larger complete systems and to the complexity of metabolic cascade reactions. Based on the fusing maps of comprehensive serum metabolome, fatty acid and amino acid profiling, we identified 35 potential metabolic biomarkers for ischemic stroke. Our analyses revealed 8 significantly altered pathways by MetPA (Metabolomics Pathway Analysis, impact score >0.10) and 15 significantly rewired modules in a complex ischemic network using the Markov clustering (MCL) method; all of these pathways became more homologous as the number of overlapping nodes was increased. We then detected 24 extensive pathways based on the total modular nodes from the network analysis, 12 of which were new discovery pathways. We provided a new perspective from the viewpoint of abnormal metabolites for the overall study of ischemic stroke as well as a new method to simplify the network analysis by selecting the more closely connected edges and nodes to build a module map of stroke.

Cerebral ischemia is one of the most devastating neurological conditions, with an approximate mortality of 5.5 million persons annually and a loss of 44 million disability-adjusted life years worldwide^1^. Although inflammatory and immune responses play important roles in the course of ischemic stroke^2^,^3^, it has been estimated that the etiology and pathophysiology remain unexplained in more than 40% of stroke cases. Previous studies found that activation of the Raf/MEK/ERK/p90RSK cascade^4^, the extracellular signal-regulated protein kinase cascade^5^, Hsp^6^, the MEK-ERK-p90RSK cascade^7^, or the ASK1/JNK cascade^8^, co-activation of the GABA A and GABA B receptors^9^ or inhibition of the MLK3/JNK3 cascade^10^ might mediate neuroprotective effects. However, these prior studies mainly focused on one or two specific pathways, i.e., the signaling of a particular protein kinase or the co-activation of certain receptors. To some extent, these results addressed only a part of the biochemical events underlying ischemic stroke.

Metabolomics is a systemic global quantitative assessment for the comprehensive analysis of changes in endogenous metabolites in a biological matrix, which can be directly coupled to biological phenotypic responses to a disease^11^,^12^, drug treatment^13^,^14^ or intervention^15^. Based on metabolite perturbations following a stroke, one can map all of the pathways related to ischemic stroke; understanding these pathways is among the key challenges facing cerebral ischemia research. Recently, several metabolomic studies have shed some light on this problem through the analysis of metabolomic profiles. For example, the levels of glutamate, glutamine, aspartate, γ -aminobutyrate (GABA), taurine, malate, fumarate, acetate, phosphocreatine, and inosine, hypoxanthine, xanthine, and uracil significantly decreased after middle cerebral artery occlusion (MCAO), as detected by high-resolution nuclear magnetic resonance (NMR) spectroscopy^16^. Additionally, metabolic patterns in the plasma and urine from patients with cerebral infarctions could be characterized by the ^1^H-NMR metabolomics approach^17^. However, at this stage, how many metabolites are involved in the current ischemic network remains unclear because most of these previous studies analyzed targeted metabolites as individual markers or pathways. Due to the complexity of diseases, with multiple metabolites distributed on different levels of signal transduction pathways, the novel approaches of systems biology, network pharmacology, modular pharmacology and bioinformatics can be employed to explore complex disease mechanisms and to design rational therapies for stroke patients^18^,^19^,^20^. Our study creatively integrated non-targeted and targeted metabolomic results to detect all of potential biomarkers that may be useful for the early diagnosis of ischemic stroke. Furthermore, we provide a framework of pathways and modules to explain the biochemical mechanism of ischemic stroke.

## Materials and Methods

### Animal blood sample collection

Six-week-old male Sprague-Dawley (SD) rats were purchased from the China Academy of Military Medical Science (Peking, China) (see Fig. S1 for the flow chart of the study). Stroke was induced using the MCAO procedure, with 1 hour of occlusion followed by reperfusion. The animals were then assessed for motor deficits, and inclusion/exclusion criteria were applied. We drew 5 ml blood from the abdominal aorta on the 3^rd^ day; we then placed 2 ml of this blood into an EDTA tube, fully mixed the sample, centrifuged the sample at 3,000 rpm for 10 min, and extracted 500 μL plasma. The remaining 3 mL blood were stored at 4 °C for 60 min and then centrifuged at 3,000 rpm for 10 min, and 1000 μL of the supernatant was collected as the serum sample. All of the samples were stored in a −80 °C freezer for further analysis.

The protocol was approved by the Ethics Committee of China Academy of Chinese Medical Sciences and was implemented in accordance with provisions of the Declaration of Helsinki and Good Clinical Practice guidelines. The investigation was conducted in accordance with the ethical principles of animal use and care.

### Serum metabolome

#### Sample preparation

Thawed plasma samples (200 μL) were mixed with acetonitrile (400 μL) by vortexing for 30 s, stored at 4 °C for 60 min, and centrifuged at 12,000 rpm for 10 min. Then, 500 μL of the supernatant was collected for freeze drying. The lyophilized sample was re-dissolved in 200 μL acetonitrile/water (1:1, v/v), centrifuged at 12,000 rpm for 5 min, and then filtered through a syringe filter (0.22 μm) for UHPLC MS analysis.

### UHPLC-TOF MS analysis

#### Chromatographic condition

Serum metabolomics was performed with UHPLC Q-TOF/MS. Chromatography was conducted using a Zorbax Eclipse plus C18 column (150 mm × 3 mm, i.d.1.8 μm, U.S., Agilent Technologies). The binary mobile phase was composed of phase A (water with 0.2% methanoic acid) and Phase B (acetonitrile). The gradient for the serum sample was 0 ~ 25 min, 0 ~ 100% B. The proportion of phase B returned to 5% in 1 min, and the column was allowed to re-equilibrate for 5 min before the next injection. The flow rate was 0.5 mL/min, and 1 μL was injected into the column. The column temperature was maintained at 40 °C.

#### TOF MS condition

MS analysis was conducted using a QTOF 6520 (Agilent, Santa Clara, USA) operating in positive (ESI +) electrospray ionization mode. Nitrogen was used as the dry gas, the cone gas flow was maintained at 10.0 L/min, and the desolvation temperature was set at 325 °C. The atomization gas pressure was set at 50 psig, the capillary voltage was set at 4000 V, the cone voltage was set at 65 V, the OCT 1 RF Vpp was set at 750 V, and the fragmentor voltage was set at 175 V. The TOF data were collected from 100–1000 m/z. The UHPLC Q-TOF/MS analysis platform provides the retention time, the precise molecular mass, and MS/MS data for the structural identification of biomarkers. The precise molecular mass was determined within measurement errors (<5 ppm) by Q-TOF/MS.

#### Quality control and method validation

First, we optimized the extraction method of plasma samples. We attempted two pretreatment methods, including nitrogen blow drying and freeze drying, and found that the freeze drying method was more advantageous to obtain a product closer in nature to the sample analyte. The selected solvent was simultaneously optimized; we ultimately chose the 1:1 mixed solution of acetonitrile/methanol to quantitatively analyze the samples to ensure that as more as possible metabolites were dissolved. We tried different chromatographic columns to optimize the separation conditions in experiments, eventually selecting the Zorbax Eclipse plus C18 chromatographic column (3 × 150 mm, and 1.8 μm) under positive patterns for use.

#### Enzyme-linked immunosorbent assay (ELISA)

First, we removed the kit from storage (Rat Elisa Kit (48T×1), Batch number: Lot#: 20130419, BlueGene Biotech Company, Shanghai, China) and let it sit in a 20−25 °C environment for 30 min. Then, on the enzyme-labeled plate, we added 50 μL standard solution into each of the blank micro-wells, according to the standard order. We then added 50 μL of standard solution into the blank micro-well or 50 μL distilled water into the blank contrast well. Subsequently, we added 100 μL enzyme marker solution to each well (excluding the blank contrast well). After sealing the enzyme mark plate with glue, we incubated the plate at 37 °C for 60 min. During the experiment, we washed the enzyme marker plates 3−5 times to ensure that the wells were filled with adequate water pressure. Finally, we added 50 μL stop solution into each well to terminate the reaction.

Instrument: ELx800 ELIASA, BioTek Co., USA.

### Pathway and network analysis

The construction, interaction and pathway analysis of potential biomarkers were performed with MetPA (http://metpa.metabolomics.ca), and database sources, including the KEGG, the Human Metabolome database, and METLIN, were used to identify the related metabolic pathways. The possible biological roles were evaluated by enrichment analysis using MetaboAnalyst. IPA used the high-quality KEGG metabolic pathways as the backend knowledge base. The impact-value threshold calculated from pathway topology analysis was set as 0.10, and those above the threshold were screened out as potentially significant pathways.

We used Cytoscape, an open source software, to integrate biomolecular interaction networks with high-throughput expression data into a unified conceptual framework. We mainly used Metscape, which is a plug-in for Cytoscape, to visualize and interpret metabolomic data in the context of human metabolic networks^21^. Metscape takes advantage of VizMapper in Cytoscape to create the Metscape visual style.

### Module analysis by Markov clustering

MCL-edge is dedicated to analyzing very large networks, scaling up to millions of nodes and hundreds of millions of edges. It comprises a small set of tools supporting algorithms that both are commonly used and scale well. The tools are ready-to-run and command-line based, often allowing multi-processing and job dispatching. Significant modules were defined when peak values of modularity and entropy were both achieved.

## Results

### Evaluation of ischemic models

**Figure 1:**
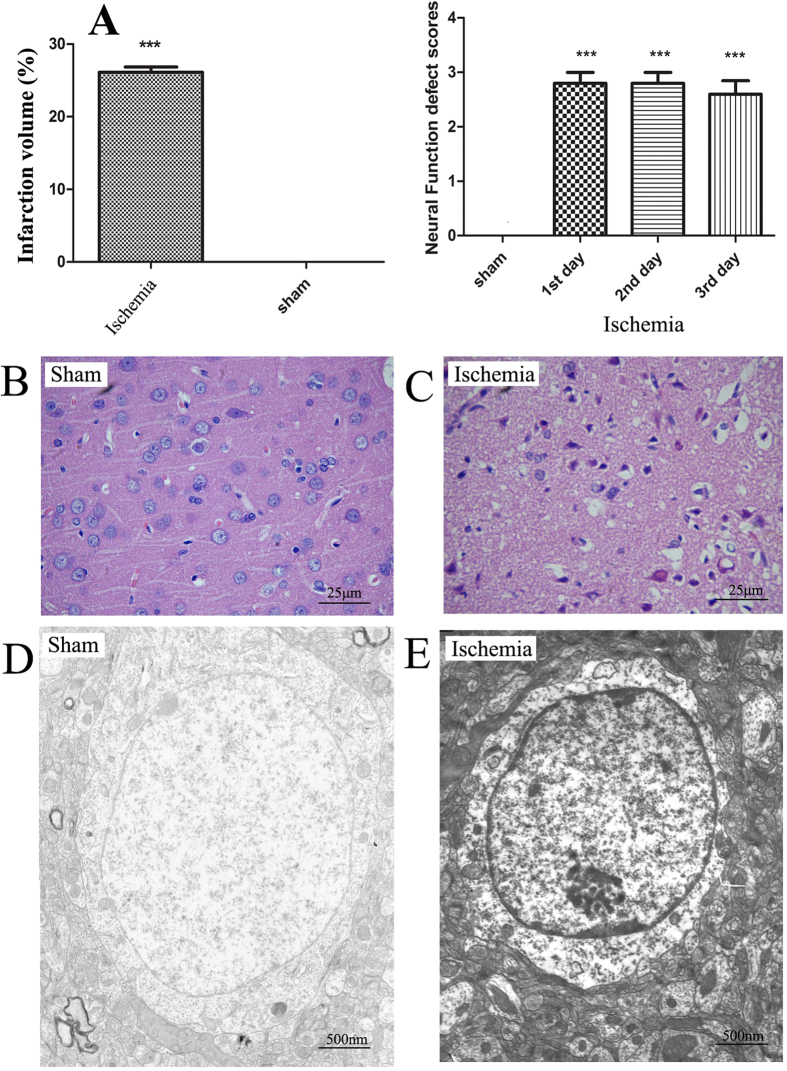
Phenotypes alteration of ischemic rats compared with sham-operated rats. (**A**) The average brain infarction volume and neurological deficits scores of the sham and ischemia groups. Data are presented as mean ± SEM. ***P < 0.001 as determined by Bonferroni corrected t tests. (**B**,**C**) The HE stains of cortical neurons of the sham and ischemia groups, respectively. (**D**,**E**) The electron microscope of cortical neurons of the sham and ischemia groups, respectively.

Ten rats in the ischemia group were successfully modeled. After 3 days, 5 rats in the ischemia group survived, and their blood samples were therefore collected for the subsequent metabolic network analysis. Meanwhile, 10 rats in the sham group were taken as control samples. The infarct volume (p < 0.001) (see Fig. S2 for TTC result) and neurological deficit score over three days (p < 0.001 on each day) after the induction of ischemia were significantly increased (Fig. 1A). Compared with the cortical tissue of sham-operated animals (Fig. 1B), significant necrosis of the cortical neuronal cells in the core of the infarct was observed in the ischemia group by hematoxylin-eosin (HE) staining, with gap-like structured neurons and enhanced basophilic staining in the cytoplasm; the neuronal cell body was deformed, and the nucleus was condensed, dissolved or disappeared (Fig. 1C). Nerve cell swelling and degeneration, cytoplasmic edema, intracellular component degradation, nuclear condensation, nuclear membrane thickening, and nuclear chromatin aggregation into blocks were observed under ischemic conditions by electron microscopy (Fig. 1E), whereas normal nerve cell structure, abundant cytoplasm, normal mitochondria and endoplasmic reticulum, abundant glycogen granules, normal cell nuclei, double-layered nuclear membranes, and evenly distributed, nuclear staining quality were observed under normal conditions (Fig. 1D). Under ischemic conditions, marked edema was noted in the cytoplasm, the amount of organelles significantly decreased, the envelope and cristae of most mitochondria became fused and obscure, and the rough endoplasmic reticulum mildly expanded, with evident fusion and degranulation.

### Identification of potential biomarkers

#### Potential biomarkers of comprehensive metabolomics

**Figure 2:**
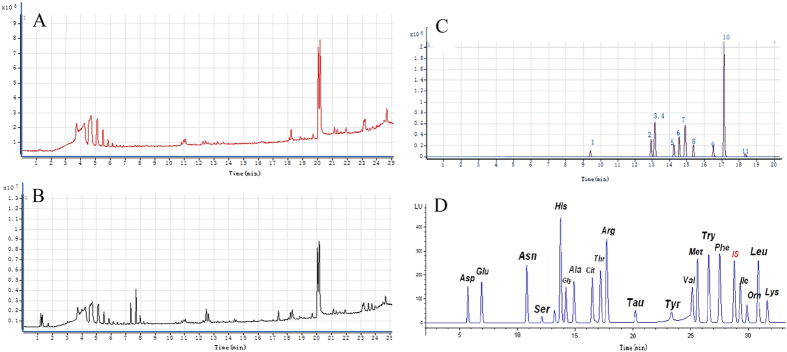
(**A**) Total ion current chromatography (TIC) of plasma samples derived from the sham group; (**B**) Total ion current chromatography (TIC) of plasma samples derived from the ischemia group; (**C**) Profile of HPLC chromatogram of serum fatty acids in ischemic rats; (**D**) Profile of HPLC chromatogram of serum amino acids in ischemic rats.

**Figure 3:**
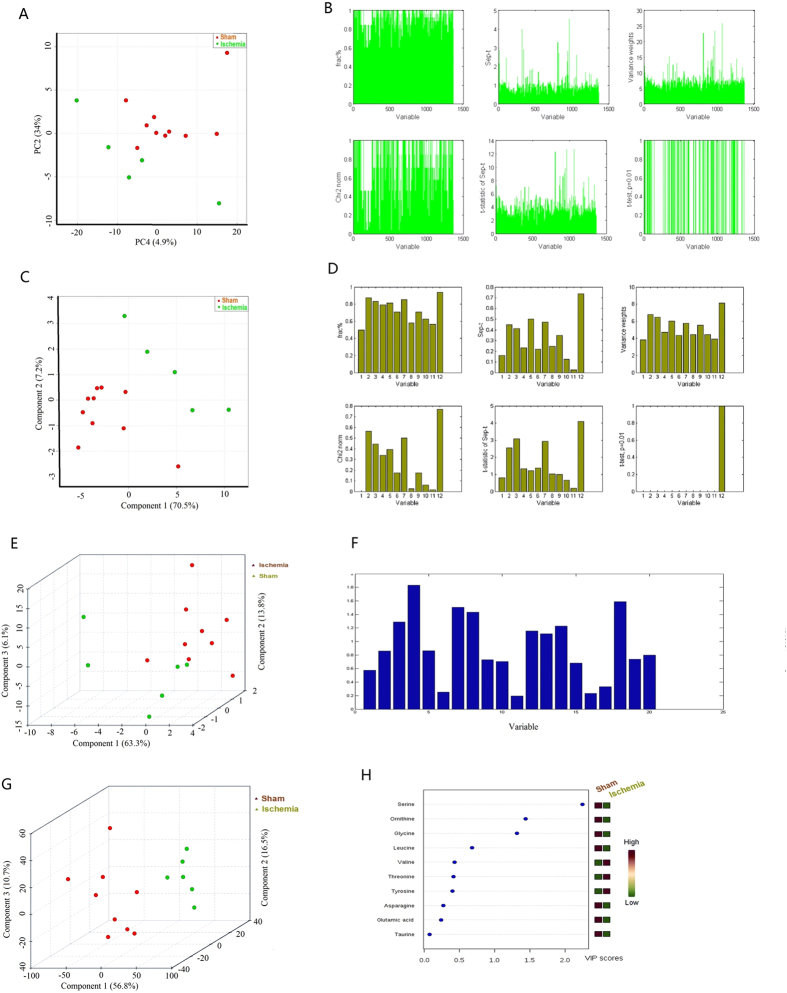
Comprehensive metabolomic, HPLC chromatogram of serum fatty acids and amino acids profiling of plasma samples from ischemic and sham-operated rats. (**A**) PCA score plots for comprehensive metabolomic data of the sham and MCAO rats (**B**) Bar plot of graphical index of separation (GIOS) of metabolomic profiling variables from the plasma samples of the sham and ischemia groups (**C**) Score plots of the PLS model of the sham and ischemia groups. The explained variance of each PC is shown in the corresponding diagonal cell (**D**) Bar plot of graphical index of separation (GIOS) of serum fatty acids from the samples of the sham and ischemia groups. (**E**) 3D score plot of the PLS model of the sham and ischemia groups (serum fatty acids). The explained variances are shown in brackets. (**F**) Bar plot of graphical index of separation (GIOS) of serum amino acids from the samples of the sham and ischemia groups (**G**) 3D Score plot from the PLS model of the serum amino acids from the sham and ischemia groups. (**H**) Important features of serum amino acid variables identified by PLS-DA. The colored boxes on the right indicate the relative concentrations of the corresponding metabolites in the sham and ischemia groups.

The total ion current chromatogram (TIC) of plasma samples derived from the sham and ischemia groups showed significant differences in metabolite abundance (Fig. 2A,B), which might contribute to the distinct separation between the sham and MCAO rats in principal component analysis (PCA) score plots (Fig. 3A). The potential biomarkers were discovered by Graphical Index of Separation (GIOS) (Fig. 3B) and were employed to build a PLS-DA model for sham and MCAO rats (Fig. 3C). The PLS score plots revealed various metabolites that could be responsible for the separation; thus, these metabolites were viewed as potential biomarkers. Finally, potentially significant biomarkers were characterized in ischemic rats (Table 1), including 15 significantly increased and 7 decreased metabolites in the ischemia group compared with the sham group.

The typical chromatogram of serum fatty acid in MCAO rats demonstrated the peak area and corresponding retention time using ultra-performance liquid chromatography/ electrospray ionization tandem mass spectrometry (UHPLC/ESI-MS/MS) for 12 free fatty acids (FFAs) (Fig. 2C); satisfactory separation of the FFAs in the mixed standard solution was achieved within 20 min. According to frct% and its χ^2^ test, the separation score (sep-t) with its *t*-test (t-statistic of sep-t) were computed for discriminating between the ischemic and sham groups (Fig. 3D). Three accordant variables (C18:3, C16:0 and C20:0) had the greatest contributions in discriminating the MCAO rats from sham controls (Fig. S1A). After selecting out these potential variables, we built a PLS-DA model for MCAO rats and sham controls, which could be clearly separated using the 3 variables selected by GIOS (Fig. 3D). The PLS score plots and loading plot further revealed distinct separation between the two groups (Fig. 3E and Fig. S3). The three fatty acids were regarded as the potentially significant biomarkers in ischemic rats (Table 2), indicating that arachidonic acid (C20:0) was significantly decreased in the ischemia group compared with the sham group, while gamma-linolenic acid (C18:3) and palmitic acid (C16:0) were significantly increased. Palmitic acid increases the risk of developing cardiovascular diseases, gamma-linolenic acid induces apoptosis and lipid peroxidation, and more importantly, arachidonic acid metabolism plays specific roles in the regulation of cerebral blood flow; the accumulation of these fatty acids leads to significant variations in the PG concentrations in brain tissues^14^.

#### Potential biomarkers of serum amino acids

Serum samples collected from the ischemic rats and sham controls contained approximately the same amino acids (Fig. 2D). The main difference was quantitative, i.e., the amino acid level in the ischemia group was significantly higher than that in the sham group. The levels of 10 of 20 amino acids (Fig. 3F,H) were significantly different (P < 0.01) between the two groups (Fig. 3G); thus, the 10 potentially significant biomarkers were characterized in the ischemic rats (Table 3). The levels of glutamic acid, threonine, valine and leucine significantly increased in the ischemia group compared with the sham group, while the levels of asparagine, serine, glycine, taurine, tyrosine and ornithine significantly decreased.

### Pathway and modular analysis

#### Metabolic ischemic pathways

Metabolic pathway analysis with MetPA revealed that 35 metabolites (Table 1, 2 and 3) significantly varied following ischemic stroke (Table S1). Eight significant metabolic pathways out of a total of 27 pathways (impact score >0.10) were found to be uniquely affected in the MCAO ischemic rats (Table 4), including VLI biosynthesis (impact score 0.67) and GST metabolism (Fig. S4; impact score 0.53), among others. Our study differed from the common pathway analysis framework such as elementary flux modes and extreme pathways in that we selected specific pathways according to the metabolic profiles of our biological samples and arranged them by importance. This pathway analysis method has practical significance for the comprehensive assessment of biochemical reactions and the related specific metabolic functions after cerebral infarction.

#### Construction of ischemic network and modular analysis

A disease network model can express the complex relationship between drugs, target and disease; nodes represent entities such as genes, proteins, small molecules, drugs, and diseases, and edges represent the interaction between nodes. We further reconstructed ischemia-related metabolic networks based on all of the relevant metabolites identified by Metscape. The whole metabolic network included 189 nodes (167 main compounds) and 237 edges (208 main reactions), involving 23 pathways in the same database (Fig. S5, Table S2).

**Figure 4:**
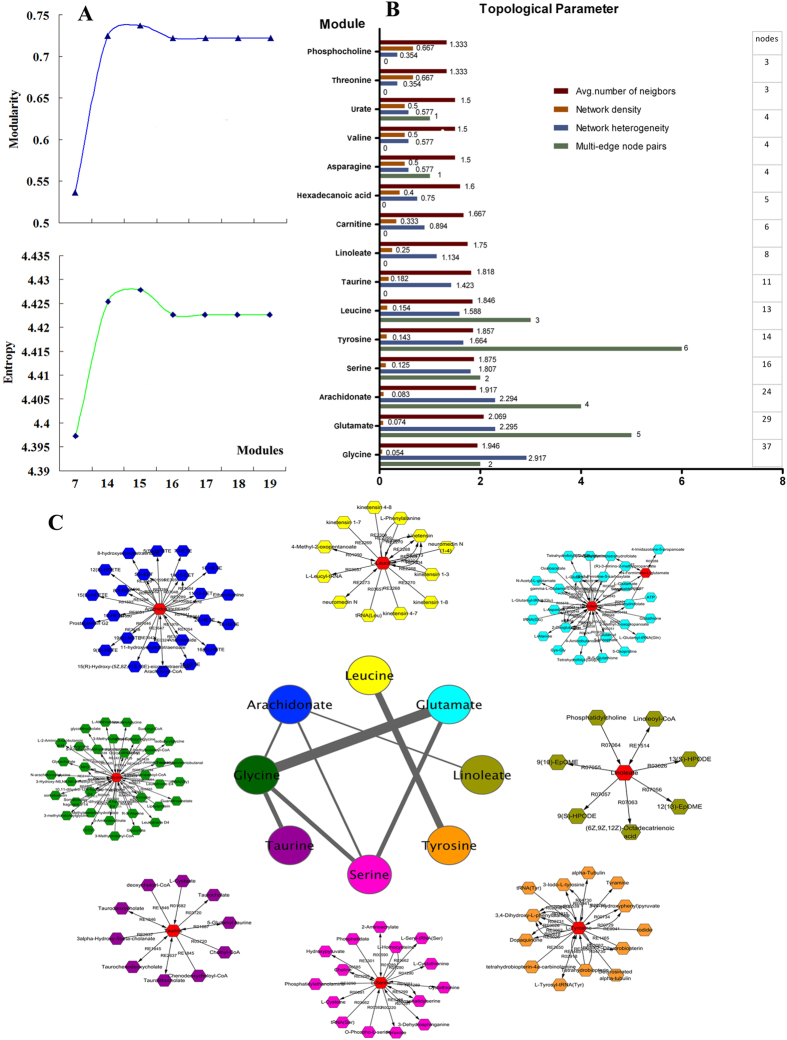
Module topological parameters and interactions. (**A**) Significant modules were defined by analysis of modularity and entropy. When values of modularity and entropy achieved vertex, 15 modules were defined. (**B**) Comparing the topological parameters of all modules by MCL method. (**C**) Visualizing the modular map by Metscape to observe the connections among 8 modules. Each color of the node represents a module, and the edge between them represents the interaction of nodes among the modules. According to different widths, these edges indicate 1, 2, 3 and 5 interactions, respectively. Outside this central map are the specific modules for each colored nodes, and the red nodes are the core nodes for the module.

The evidence shows that the network of biological systems has the module structure, indicating that some molecules in the network perform some types of biological function. We then divided the constructed metabolic network into 15 different modules by MCL when the maximum values of modularity and entropy were both achieved (Figs 4A, S6). Then, we computed the topological parameter for these modules by MCL to observe the structure differences among the 15 modules (Fig. 4B). The average number of neighbors ranged from 1.33 (threonine and phosphocholine modules) to 2.069 (glutamate module), while the number of nodes ranged from 3 (phosphocholine and threonine modules) to 37 (glycine module). The network density was largely changed, from 0.054 (glycine module) to 0.667 (threonine and phosphocholine modules), and the network heterogeneity spanned from 0.354 (threonine and phosphocholine modules) to 2.917 (glycine module), as well. Most of the modules did not have any multi-edge node pairs, except the tyrosine, glutamate, arachidonate, leucine, glycine ,urate and asparagine modules, which had 6, 5, 4, 3, 2, and 1 node pairs, respectively. The value of network heterogeneity increased with the number of nodes (R = 0.9699), indicating that the topological characteristics of modules became more and more diverse when more nodes were added.

Our study demonstrated that ischemic stroke is a network phenomenon. The ischemia-related metabolic networks constructed here may be beneficial for disease classification and diagnosis and drug target candidate identification. The metabolic network was characterized by a high intrinsic potential. Our study successfully established 15 cerebral infarction modules. Module division will help to rebuild the decreased cerebral state in the course of the disease to reduce the complexity of or to shrink the disease network without the loss of information.

#### Construction of connections in the modular map

The existing connections in the 8 most closely connected modules out of 15 modules in the center of the modular map were visualized by Cytoscape (Fig. 4C). We found that the glycine and glutamate modules and tyrosine and leucine modules connected on 5 and 3 edges between them, respectively; the glycine and serine module, serine and glutamate module, and taurine and glycine module pairs each shared 2 edges (connections), respectively; however, the glycine and arachidonate module, arachidonate and linoleate module, arachidonate and serine module pairs each shared only 1 edge (connection), respectively. Although the tyrosine and leucine modules had 3 connections between them, they appeared to be outside of this central network. The module structure in the network has self similarity, with individual modules connected to the nodes of the adjacent modules in the network by bottleneck or through an inter-modular hub, resulting in overlapping edges between modules. These results suggest that further identification of clear node bottlenecks will significantly impact the effective screening of drug targets^22^.

#### Diverse metabolic modules based on similarity analysis

**Figure 5:**
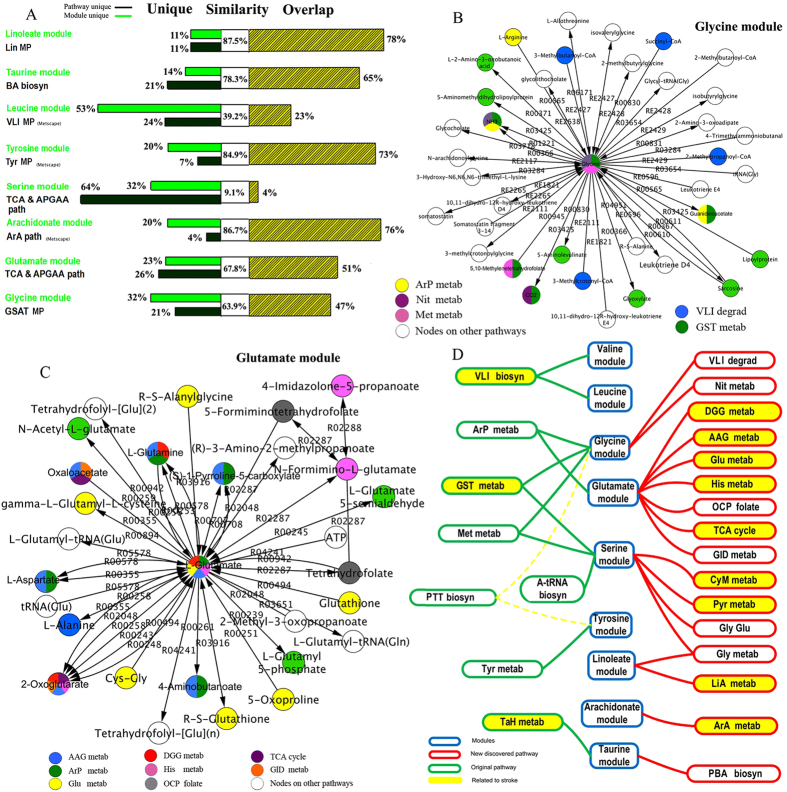
Multiple dimensional analysis of the relationship between modules and pathways. (**A**) Comparing the nodes similarity between the 8 pathways and the 8 modules structure in Metscape. Based on different contributions of overlapping and non-overlapping nodes between modules and pathways, the corresponding similarity profiles could uncover this trend. (**B**,**C**) Pathways convergence in the glycine and glutamate modules, respectively. Different colors indicate different pathways in a module. A node with two or three colors indicates two or three pathways in the same node. (**D**) Modules divergence in known and unknown pathways. The significant modules are displayed in the middle line, with 8 known pathways (shown in Fig. 4B) and 16 unknown pathways located in the left and right sides, respectively. Modules that can be verified in literature are highlighted in yellow. The PTT biosyn pathway was found to diverge into glycine and tyrosine modules.

Based on the pathway structure, the above 8 modules had some overlapping nodes with a certain pathway from the same database. We evaluated the similarity between the pathway and the module by the vectorial angle method^23^ and sequenced the similarity between these 8 pathways and modules (Fig. 5A). The similarity values for the 8 modules were 87.5% (78% identical ratio and 7 overlapping nodes, Fig. S7H), 86.72% (76% identical ratio and 19 overlapping nodes, Fig. S7C), 84.87% (73% identical ratio and 11 overlapping nodes, Fig. S7E), 78.33% (65% identical ratio and 9 overlapping nodes, Fig. S7G), 67.81% (51% identical ratio and 20 overlapping nodes, Fig. S7B), 63.94% (47% identical ratio and 22 overlapping nodes, Fig. S7A), 39.22% (23% identical ratio and overlapping nodes, Fig. S7F), and 9.13% (4% identical ratio and 2 overlapping nodes, Fig. S7D), respectively (Fig. 5A) (Table S3). It was revealed that these 8 modules not only held higher similarity dependent on increases in the overlap percentage but also preserved diverse metabolic homogeneities despite still having their own unique nodes. These results confirm that the modules in the network have the ability to be overlapped. Information exchange and transmission occur through the overlapping area in the disease network; this will be important for the further study of signal transduction, network cooperation and other behaviors in the metabolic network of cerebral infarction.

#### Hierarchical cross-talk among modules and pathways

First, we described the number of convergent and divergent pathways corresponded to each module. We then re-entered the nodes of each module into a web-based metabolomics tool (MetPA) for significant pathway analysis and visualization. As a result, 5 convergent pathways were identified in the glycine module (Fig. 5B), and 8 convergent pathways were identified in the glutamate module (Fig. 5C). Regarding the other modules, the serine, taurine, linoleate, arachidonate, tyrosine, leucine, and valine modules showed convergence in 7, 2, 2, 1, 1, 1, and 1 pathways, respectively. In contrast, 5 pathways, Met, GST, ArP metabolism and VLI and PTT biosynthesis could diverge into two modules. In total, 24 pathways converged or diverged into 9 modules based on the known databases summarized in Fig. 5D.

Then, we analyzed multiple pathway-related convergent and divergent modules. With the exception of 3 modules (taurine, tyrosine and arachidonate) other modules could converge or diverge into multiple pathways. For example, the glycine and glutamate modules converged into the ArP metabolism pathway, while the serine module diverged into 3 pathways, Met and GST metabolism and A-tRNA biosynthesis. To further explore the role of these pathways in ischemia, we verified them using a data mining method. In addition, 3 of 8 pathways extracted from MetPA and 9 of 16 pathways identified in this study (total of 50%) could be verified in the literature (Fig. 5D, Table S3). The module can be viewed as a subnetwork with a similar function that can include multiple pathways. Simple pathway analysis often neglects the natural organizing principle of the network. Given the complexity and the lacunas present in its structure, an operational alternative is to work with a simplified model network. Different levels of network comparison may uncover novel functions and disease-specific changes, thereby aiding drug design^24^.

### Validation by ELISA and other independent experiments

**Figure 6:**
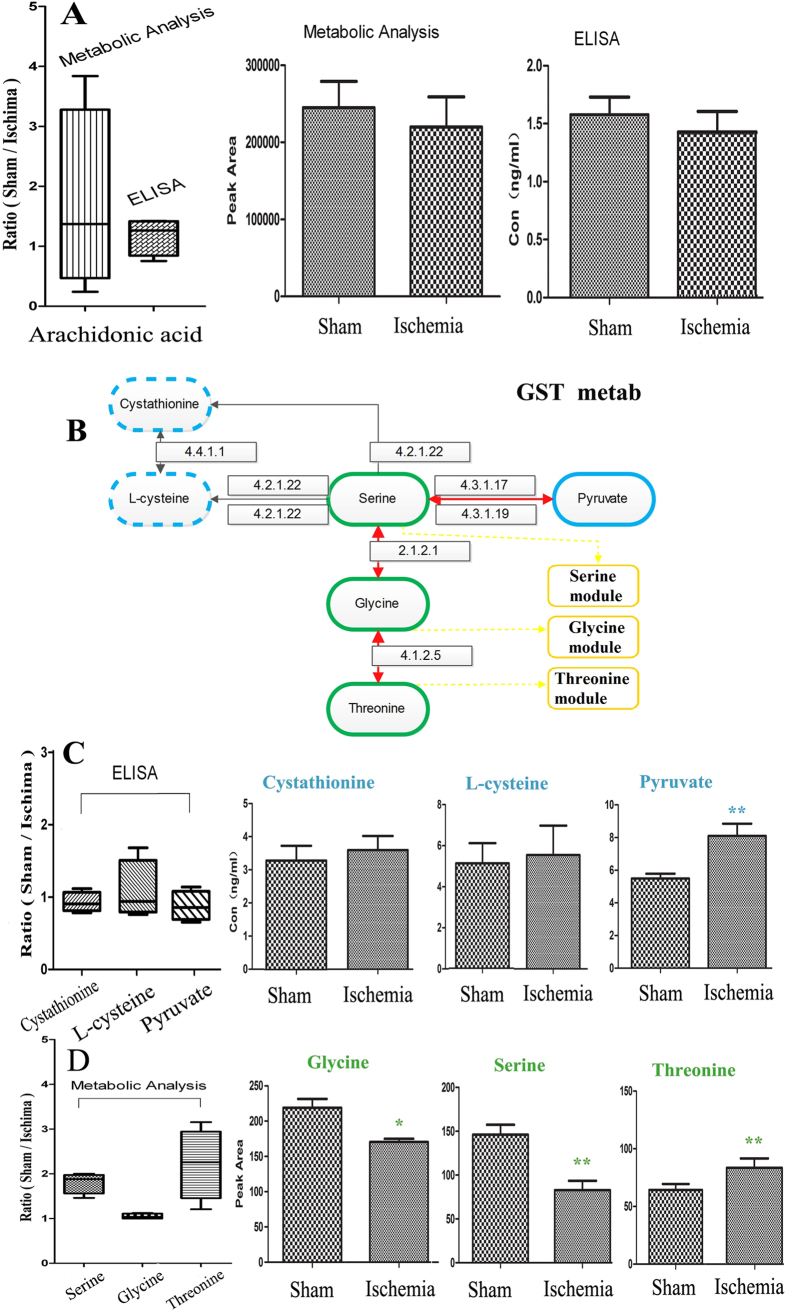
Validated metabolites, pathways and modules using independent experiments. (**A**) Box plot comparing the concentrations of arachidonic acid (sham/ischemia groups) by metabolic analysis and ELISA test. n = 3. (**B**) Map of the GST Metab on KEGG. (**C**) Box plot comparing the concentrations of cystathionine, L-cysteine and pyruvate (sham/ischemia groups) by ELISA test. n = 3. (**D**) Box plot comparing the concentrations of glycine, serine, threonine (sham/ischemia groups) by metabolic analysis. n = 3. Data are presented as mean ± SEM. *P < 0.05, **P < 0.01 as determined by Bonferroni corrected t tests. n = 3.

To verify the pathways and modules mentioned above, we selected 4 nodes from 2 pathways for ELISA to identify the variation between the sham and ischemia groups. These nodes contained arachidonate from the arachidonic acid metabolism pathway (Fig. 6A) and cystathionine, L-cysteine and pyruvate, glycine, serine, and threonine from the GST MP_(Metscape)_ (Fig. 6B). The results of arachidonic acid by the two metabolic analysis methods (P = 0.6928) were consistent with those by ELISA (P = 0.6404); the GST MP_(Metscape)_ was activated in the ischemic process, manifesting as an increase in pyruvate (P = 0.0024) by ELISA (Fig. 6C) and reductions in glycine (P = 0.0245), serine (P = 0.0055) and an increase in threonine (P = 0.0316) by metabolic analysis (Fig. 6D); the levels of cystathionine (P = 0.666), and L-cysteine (P = 0.8014) were not significantly altered.

## Discussion

Brain ischemia is a process of delayed neuronal cell death, not an instantaneous event^25^. The development of an ischemic event, whether silent or painful, represents the cumulative impact of a sequence of pathophysiological events^26^. In particular, biomarker discovery for the early diagnosis of ischemic stroke will provide the opportunity to modify lifestyle and permit timely pharmacological treatment. In this study, comprehensive plasma metabolic profiling was performed with UHPLC-Q/TOF-MS and multivariate statistical analysis. As a result, 22 specific metabolites that might be helpful to discover potential individuals requiring treatment for cerebral ischemia were identified. We also conducted serum fatty acid and amino acid profiling, respectively, and found 3 fatty acids and 10 amino acids that might be associated with ischemic cascade. As the comprehensive plasma metabolite profiling usually reveals abundant chemicals in biological samples, it cannot detect the chemicals at low concentrations, to some extent. Based on comprehensive plasma metabolic profiling, we found that some amino acids and fatty acids might be related to ischemia, but such profiling cannot provide information on metabolites other than the targets. Thus, we combined the untargeted and targeted metabolomic method, and identified 35 metabolites that were potential ischemic stroke biomarkers. The analyses provided a differential metabolic profile between ischemic and sham-operated rats, which may be correlated with metabolic phenotypes. In addition to the central nervous system and anti-inflammatory functions, most metabolites have some connections to cerebral ischemic stroke progression.

### Newly discovered metabolites may deepen our knowledge of the metabolic mechanism of ischemia

It has been shown that creatine supplementation has some positive effects on the central nervous system^27^, and creatine-derived compounds may reproduce the neuroprotective effects of creatine while better crossing the neuronal plasma membrane and the blood-brain barrier^28^,^29^. An increased uric acid level was found to be associated with a decreased risk of poor outcomes among 3,231 patients with acute stroke^30^ and silent brain infarction^31^,^32^. Prosopinine has an impact on the central and autonomic nervous systems^33^. Plasma lysophosphatidylcholine levels are reduced in obesity and type 2 diabetes^34^, and can induce caspase-3-dependent endothelial cell death^35^,^36^ and atherosclerotic plaque inflammation in humans^37^. The administration of acetyl-L-carnitine (ALCAR) attenuates neuronal damage, prevents apoptosis, and improves the energy status in hypoxic stress via mechanisms that are less understood^38^. Pre-treatment with chronic ALCAR significantly reduced the infarct size^39^, and post-ischemic treatment with ALCAR improved early clinical recovery and prevented significant weight loss in rat models of focal cerebral ischemia^40^. Gamma-linolenic acid induces apoptosis and lipid peroxidation^41^ and is also thought to be an anti-inflammatory fatty acid^42^. Neurochemical monitoring has indicated that hypothermia decreases glutamate, glycerol, lactate, and pyruvate in the “tissue at risk” area of the infarct but not within the infarct core^43^. Arachidonic acid metabolism plays specific roles in the regulation of cerebral blood flow (CBF)^44^,^45^, the modulation of vascular permeability, and the modulation of excitatory and inhibitory neurotransmitter release^46^,^47^. Previous studies of arachidonic acid metabolism in experimental cerebral ischemia and reperfusion^48^,^49^,^50^ have shown that the accumulation of fatty acids, mainly arachidonic acid, due to the breakdown of structural membrane lipids leads to significant variations in prostaglandin (PG) concentrations in brain tissues^48^,^51^. Once free, arachidonic acid undergoes both enzyme-independent and enzyme-mediated oxidative metabolism, resulting in the formation of a number of biologically active metabolites, which themselves contribute to pathological stroke outcomes^52^. The relationship between dietary omega-3 fatty acids and the risk of developing CVD began to emerge in the late 1970s^53^,^54^. Moreover, these results suggest that glycerophospholipid metabolism, particularly phosphatidylcholine biosynthesis, plays an important role in ischemic stroke. Excessive glutamate release and impaired uptake occur as part of the ischemic cascade and are associated with stroke^55^. The serine metabolism pathway might be associated with the incidence of cerebral ischemia^56^. Taurine crosses the blood–brain barrier and has been implicated in a wide array of physiological phenomena^57^. In conclusion, our study found that the levels of 35 metabolites, most of which are reported to relate to stroke or to have neuroprotective functions, were significantly changed after the onset of ischemic stroke. This result differs from that reported by Yun Wang *et al.*^58^. These 35 metabolites can be considered as potential biomarkers of cerebral infarction and could be beneficial for early stage disease risk identification, early diagnosis, pathological mechanism research and drug target screening.

### Network and modular analyses may contribute to future metabolic research

Complex diseases or complex physiological processes have been described as network phenomena for quite some time^59^,^60^,^61^. The disease-related molecular networks are no doubt helpful to predict novel disease biomarkers by using network-assembled bio-data, including novel disease-related genes, proteins or metabolites. In this study, we used MetPA and Metscape software to analyze stroke-related pathways and to construct a compound network map for further studies. Among 24 pathways, 12 pathways were shown to be related to cerebral ischemia, and the other 12 had never been previously reported.

Metabolic pathways are a series of chemical reactions occurring within a cell; each pathway is modified by a series of chemical reactions and is mainly related to a certain biological function. A module is the minimum functional unit in a pharmacological profile. Disease can be viewed as a breakdown of the robustness of normal physiological systems and the re-establishment of robust and potentially progressive disease^62^. We define an active unit as a module to describe the sub-networks. Pathway analysis of central catabolism is feasible to assess network properties such as flexibility and functionality. Metabolic pathway analysis is important for assessing inherent network properties in reconstructed biochemical reaction networks^63^. However, most concept for pathway analysis rely on one common framework such as elementary flux modes and extreme pathways. In fact, the metabolic network can be subdivided into several small, highly connected functional units, termed metabolic modules. Each module can be observed as a discrete entity of several elementary components and performs an identifiable task, separable from the functions of other modules. Recent studies have found that metabolic networks are characterized by a high intrinsic potential modularity. Network size markedly determines modular organization; the larger the network, the greater the modularity. Moreover, modularity is a driving force for the self-organization of networks and is a basis for functional adaptation^64^,^65^. The unique modular structure facilitates participation in both system-wide and pathway-specific regulatory processes^66^. In our study, we successfully built a network for cerebral ischemia and divided the network into 15 modules. Meanwhile, we verified these results by ELISA, confirmed these modules by testing the central points, confirmed the consistency of the ELISA and metabolic analysis results by testing the same compound independently, and confirmed the existence of these significant pathways by testing more points in the same pathway.

The significance of this research includes the following: (1) we creatively integrated the non-targeted and targeted metabolomic results to detect all of the potential biomarkers that may be useful for the early diagnosis of ischemic stroke; (2) the network map provided a new perspective from the viewpoint of abnormal metabolites for the overall study of ischemic stroke and offered a new method for further studies of the stroke-related network; (3) in addition to constructing a complicated network map, we also provided a method (modular pattern) to simplify the map by selecting the more closely connected edges and nodes to build a module map for stroke; and (4) we discovered 12 new pathways based on modularity of the metabolic network and found that most of the new pathways were related to stroke, indicating that more modules of the network may be somewhat related to ischemia. Further studies will be required to shed more light on the actual contribution of these metabolic pathways and modules that are related to cerebral ischemia.

In brief, our study found glutamic acid, serine, asparagine, glycine, taurine, tyrosine, valine, ornithine, gamma-linolenic acid, arachidonic acid, palmitic acid, creatine, uric acid, phytosphingosine, L-carnitine, glycerophosphocholine, lysoPE (18:0/0:0) and lysoPC (18:1(9z)) may be potential biomarkers for ischemic stroke. These metabolite biomarker can be used for early stage disease risk identification, early diagnosis, pathological mechanism research and drug target screening. Additionally, the result indirectly confirmed that the module structure in the network has self-similarity and overlapping ability. The module can be viewed as a sub-network with a similar function that includes multiple pathways. Different levels of network comparison may uncover novel functions and disease-specific changes, thereby aiding drug design.

## Additional Information

**How to cite this article**: Zhu, Y. *et al.* System-wide assembly of pathways and modules hierarchically reveal metabolic mechanism of cerebral ischemia. *Sci. Rep.* **5**, 17068; doi: 10.1038/srep17068 (2015).

## Supplementary Material

Supplementary Information

## Figures and Tables

**Table 1:** Potential biomarkers of ischemic stroke identified from comprehensive metabolomic profiling.

Metabolites	RT(min)	m/z	Actual mass	Formula	Fold
Creatine	1.33	132.0716	131.0695	C_4_H_9_N_3_O_2_	1.29↑
Uric acid	1.57	169.0268	168.0283	C_5_H_4_NO_4_N_3_	1.35↑
Phytosphingosine	0.97	318.2006	317.293	C_18_H_39_NO_3_	0.79↓
LysoPC(16:1)	16.58	494.3251	493.3168	C_24_H_48_NO_7_P	1.82↑
LysoPC(18:0)	21.53	524.3726	523.3638	C_26_H_54_NO_7_P	1.01↑
L-Valine	1.30	118.0863	117.0790	C_5_H_11_NO_2_	1.64↑
L-carnitine	1.28	162.1120	161.1052	C_7_H_15_NO_3_	1.40↑
L-acetylcarnitine	1.60	204.1218	203.1158	C_9_H_17_NO_4_	0.66↓
Glycerolphosphocholine(16:0)	18.37	497.3444	496.341	C_8_H_20_NO_6_P	1.38↑
13S-Hydroxyoctadecadienoic acid	21.95	297.2408	296.2351	C_18_H_32_O_3_	0.99↓
Tyr Val/Val Tyr	19.70	281.1496	280.1423	C_14_H_20_N_2_O_4_	1.28↑
6-hydroxysphingosine	18.70	316.2846	315.2773	C_18_H_37_NO_3_	1.93↑
Prosopinine	15.83	288.2533	287.2460	C_16_H_33_NO_3_	0.72↓
(3R)-3-isopropenyl-6-oxoheptanoic acid	19.70	185.1172	184.1099	C_10_H_16_O_3_	0.89↓
cis-4-Decenoic acid	21.77	171.1380	170.1307	C_10_H_18_O_2_	0.93↓
Eicosapentaenoyl Serotonin	23.15	461.3163	460.3090	C_30_H_40_N_2_O_2_	0.93↓
LysoPC(22:6(4z,7z,10z,13z,16z,19z)	17.32	568.3398	567.3325	C_30_H_50_NO_7_P	1.44↑
LysoPE(18:0/0:0)	17.05	482.3231	481.3168	C_23_H_48_NO_7_P	2.53↑
LysoPE(0:0/20:0)	19.85	510.3520	509.3481	C_25_H_52_NO_7_P	1.75↑
LysoPC(18:1(9z))	19.07	522.3555	521.3481	C_26_H_52_NO_7_P	1.95↑
LysoPc(16:0)	18.37	496.3399	495.3325	C_24_H_50_NO_7_P	1.37↑
LysoPC(20:4)	17.40	544.3406	543.3325	C28H50NO7P	1.39↑

Arrow indicates significantly up-regulated or down-regulated metabolites in the ischemia group compared with the sham group.

**Table 2:** Potential biomarkers of ischemic stroke identified from fatty acid profiles.

Fatty acid	RT(min)	Abbrev	Formula	m/z	Fold
γ-linolenic acid	13.00	C18:3	C_18_H_30_O_2_	278.22458	1.21↑
Arachidic acid	14.57	C20:0	C_20_H_40_O_2_	312.3028	0.89↓
Palmitic acid	16.50	C16:0	C_16_H_32_O_2_	256.2402	1.39↑

Arrow indicates significantly up-regulated or down-regulated metabolites in the ischemia group compared with the sham group.

**Table 3:** Potential biomarkers of ischemic stroke identified from amino acid profiles.

*Amino acid*	*RT(min)*	*Exact mass*	*Relative peak area*	*Formula*	*Fold*
Glutamic acid	7.661	147.0532	17.96268	C_5_H_9_NO_4_	1.38↑
Asparagine	10.704	132.0535	4.35813	C_4_H_8_N_2_O_3_	0.58↓
Serine	11.709	105.0426	86.39092	C_3_H_7_NO_3_	0.56↓
Glycine	14.557	75.032	77.44959	C_2_H_5_NO_2_	0.88↓
Threonine	18.055	174.1117	35.54111	C_6_H_14_N_4_O_2_	1.23↑
Taurine	20.966	125.1457	55.17722	C_2_H_7_NO_3_S	0.96↓
Tyrosine	23.886	181.0739	13.36174	C_9_H_11_NO_3_	0.65↓
Valine	26.357	117.079	116.29909	C_5_H_11_NO_2_	1.64↑
Ornithine	31.178	132.0899	79.29524	C_5_H_12_N2O_2_	0.63↓
Leucine	31.858	131.0946	246.61391	C_6_H_13_NO_2_	1.18↑

Arrow indicates significantly up-regulated or down-regulated metabolites in the ischemia group compared with the sham group.

**Table 4:** Result for pathway analysis with MetPA.

*Pathway*	*Total*	*Expected*	*Hits*	*Impact*
VLI biosyn	11	0.22	2	0.67
GST metab	32	0.64	4	0.53
PTT biosyn	4	0.08	1	0.50
TaH metab	8	0.16	1	0.43
Met Metab	9	0.18	2	0.40
A-tRNA biosyn	67	1.34	7	0.14
ArP metab	44	0.88	2	0.14
Tyr metab	42	0.84	1	0.14
