# Supplementary material for: System-wide assembly of pathways and modules hierarchically reveal metabolic mechanism of cerebral ischemia
Source: Sci Rep. 2015 Dec 1;5:17068. doi: 10.1038/srep17068 (PMC4664864; doi:10.1038/srep17068)
Supplement: Supplementary Information [file srep17068-s1.doc]

**System-wide assembly of pathways and modules hierarchically reveal metabolic mechanism of cerebral ischemia**

Yan Zhu^1,2,4,ξ^, Zhili Guo^1,5,ξ^, Liangxiao Zhang^6,ξ^, Yingying Zhang^1^, Yinying Chen^1^, Jingyi Nan^4^, Buchang Zhao^4^, Hongbin Xiao^3,*^, Zhong Wang^1,*^,Yongyan Wang^1,*^

^1^ Institute of Basic Research in Clinical Medicine, China Academy of Chinese Medical Sciences, Beijing, 100700, China

^2^ Beijing Electric Power Hospital, Capital Medical University, Beijing,100073, China

^3^ Beijing University of Chinese Medicine, Beijing, 100029, China

^4^ Shanxi Buchang Pharmaceutical Co.Ltd, Xi’an, 712000, China

^5^ Jiaxing Traditional Chinese Medicine Affiliated Hospital of Zhejiang Chinese Medical University, Jiaxing, 314000, China

^6^ Oil Crops Research Institute, Chinese Academy of Agricultural Sciences, Wuhan, 430062, China

^ξ^ These authors contributed equally to this study.

^*^Corresponding authors:

Prof. Hongbin Xiao, Email: [hbxiao@bucm.edu.cn](mailto:hbxiaodicp@126.com), Tel: +86 10 64035275

Prof. Zhong Wang, Email: zhonwnatrue@126.com, [Tel:+86](tel:+86) 10 64014411-3308

Prof. Yongyan Wang, Email: wangyongyan2013@126.com, [Tel:+86](tel:+86) 10 84046033

**1. Extended experimental procedures**

**2. Supplementary tables and figures**

Table S1 The known biological functions for 35 metabolites

Table S2 Compound name, synonyms, formula, 2D structure, molecular weight, CAS number and smiles string for the nodes in compound network map

Table S3 Comparing the pathways and the modular nodes

Table S4 Stroke-related pathways that can be verified from literature

Fig. S1 the flowchart of system-wide assembly of pathways and modules for Cerebral Ischemia

Fig. S2 The stroke shape and size stained with TTC

Fig. S3 Loading plot of serum fatty acid variables from PLS models of the sham and model groups

Fig. S4 Metabolic ischemic pathways analyzed with MetPA

Fig. S5 Compound network map output by the Metscape under the operation of Cytoscape. Blue hexagon is a seed

Fig. S6 Modular pattern of ischemic stroke by MCL

Fig. S7 (A) Comparing the nodes similarity between the glycine module and the glycine, serine, alanine, threonine metabolism pathways in cytoscape; (B) Comparing the nodes similarity between the glutamate module and the urea cycle and metabolism of arginine, proline, glutamate, aspartate and asparagine pathways in cytoscape; (C) Comparing the nodes similarity between the arachidonate module and the arachidonic acid pathways in cytoscape; (D) Comparing the nodes similarity between the serine module and the urea cycle and the metabolism of arginine, proline, glutamate, aspartate and asparagine pathways in cytoscape; (E) The tyrosine module has 11 overlapping nodes with the tyrosine metabolism pathway in cytoscape; (F) Comparing the nodes similarity between the leucine module and the valine, leucine and isoleucine metabolism pathways in cytoscape; (G) Comparing the nodes similarity between the taurine module and the bile acid biosynthesis pathway in cytoscape; (H) Comparing the nodes similarity between the linoleate module and the linoleate metabolism pathway in cytoscape

**1. Extended experimental procedures**

**Animals**

**Preparation of MCAO models**

Six-week-old male SD rats were purchased from China Academy of Military Medical Sciences(Beijing,China). The experimental procedures were reviewed and approved by the Animal Research Committee at XiYuan Hospital of China Academy of Chinese Medical Sciences.The rats were housed individually and fed a laboratory standard diet (Lab Rodent Chow Die 5001, Ralston Purina Co.). Animals were cared and handled according to The Guide for the Care and Use of Laboratory Animals (NIH publication, revised in 2010).

Totally 20 rats were randomly divided into two groups, i.e.the ischemia group (n=10) and the sham group (n=10). The method of Longa of bolt wire was applied with slight modification(Longa et al.1989). Briefly, we anesthetized the rats (weight, 200-230g) with 10% chloral hydrate (4mL/kg, ip),then fixed them in the supine position, disinfected their skin on a sterile towel.After a midline neck incision, the right common carotid artery (CCA), internal carotid artery (ICA) and external carotid artery (ECA) were exposed, and the proximal ECA and CCA were then ligated. A specialized nylon suture of 0.2 mm diameter was introduced from the lumen of the distal CCA just before bifurcation into the ICA until resistance was felt. Thus, the origin of the middle cerebral artery (MCA) was occluded by the nylon suture. The average depth of filament insertion was 18.5±0.5 mm away from the bifurcation. Then, the exposed vessels were carefully ligated to prevent bleeding, and the incision was closed aseptically. Rats in the sham group were subjected to the same surgical procedure, but the suture was not advanced beyond the internal carotid bifurcation. Rectal temperature of the rats was maintained at 37±0.5°C using a temperature-regulated heating pad. After revival from anesthesia, the animals were put back into cages with the room temperature maintained at 27±0.5°C. In this study. only rats that exhibited left upper limb bending, rotated to the left side while walking or with paralyzed left limbswere included.

**Evaluation of infarct volume**

At 72h after reperfusion, 5 rats in the ischemia group were sacrificed by rapid decapitation under deep anesthesia with 10% chloral hydrate.Their brains were rapidly removed , sliced into five2-mm-thick consecutive coronal sections, then stained with 2% Triphenyl Tetrazolium Chloride solution (TTC) at 37ºC for 30min in the dark, followed by fixation with 4% poly-formaldehyde solution for 24h. The slices were photographed with a digital camera and analyzed by an image processing system (AlphaEaseFC 4.0, Alpha Innotech, San Leandro, CA, USA). Infarct volume was obtained according to the indirect method proposed by Swanson et al. (1990) and corrected for edema by comparing the volume of the ischemic and non-ischemic hemispheres as described by Lin et al. (1993). We measured the infarct area for each brain slice by image analysis Luxex-F instrument, and calculated the infarctvolume according to the formula V = t (A1 + A2 + ... An) - (A1 + An ) t / 2, in which t indicates the slice thickness, and A indicates the infarct size.
**Assessment of neurological deficit score**

Neurological deficit scoring(NDS) was measured on day 1^st^, 2^nd^ and 3^rd^ after stroke but before sacrification as described previously (Kofler et al., 2006). Each rat was assessed by three examiners who were blinded to the identity of the rat or treatment protocol. The scoring system was as follows: 0, no motor deficit (normal); 1, forelimb weakness and torso turning to the ipsilateral side when held by tail (mild); 2, circling to the contralateral side but normal posture at rest (moderate); 3, unable to bear weight on the affected side at rest (severe); and 4, no spontaneous locomotor activity or barrel rolling (critical). If no deficit was observed 60 min after the beginning of the occlusion period, the animal was excluded from further study.

**Transmission electron microscopy (TEM)**

TEM was applied to evaluate the effect of these compounds on cortical neurons at the ultrastructural level. Cortical tissue fragments of rat brain were fixed with 2.5% glutaraldehyde solution at 4°C overnight and were washed with PBS after fixing with 1% osmic acid for 2 hours. Being embedded in an Epon/Araldite mixture and stained with uranyl acetate and lead citrate, the cortical neurons were observed under a 1230 type transmission electron microscope (Electron Co., Japan) and photographs were taken.

**Instruments and reagents**

Instruments: Agilent 1290 Ultra performance liquid chromatography system (Agilent Corp Germany) equipped with binary pump, automatic sampler and variable wavelength ultraviolet detector; Agilent 6520 QTOF-MS (Agilent Corp USA.com) equipped with double ESI ion source; Centrifuge and freeze drying apparatus; Agilent 1200 RRLC equipped with variable wave length fluorescence detector (American Agilent Company); Fluorescence detection wavelength of 338 nm (spectral bandwidth 10nm), and reference wavelength of 390 nm (spectral bandwidth 10 nm).

Reagents: Chromatographic grade acetonitrile, methanol and formic acid were all purchased from Burdick & Jackson (USA). N-hexane and all other solvents were of analytical grade and purchased from Tianjin Kermel Chemical Reagent Co., Ltd. (Tianjin, China). Water for HPLC separations was purified with a Milli-Q system (Millipore, Bedford, USA).

Standard substances: All the standards (> 99.0% purity) used in this experiment were purchased from Sigma (St. Louis, MO, USA), including lauric acid (C12:0), eicosapentaenoic acid (C20:5), r-linolenic acid (r-C18:3), myristic acid (C14:0), palmitoleic acid (C16:1), arachidonic acid (C20:4), linoleic aicd (C18:3), docosapentenoic acid (C22:5), palmitic acid (C16:0), oleic acid (C18:1), heptadecanoic acid (C17:0) (IS, internal standard)for fatty acid profiling analysis and aspartic acid, glutamic acid, serine, histidine,tryptophan, alanine, leucine, isoleucine, tyrosine, methionine, threonine, valine, lysine, arginine, arevaline (IS), phenylalanine, citulline, ornithine, taurine, asparagine, and glutamine for amino acid profling analysis. O-phthaldialdehyde (OPA) and 3-mercaptoethanolwrer were purchased from the Accostandard Company (USA).Phase transfer catalyst 18-Crown-6 and derivation reagent a-bromoa- cetophenone (phenacyl bromide),as well as creatine and uric acid were also purchased from Sigma. Acetonitrile of chromatographic grade was obtained from Burdick & Jackson (USA). Other solvents were of analytical grade and purchased from Tianjin Kermel Chemical Reagent Co., Ltd. (Tianjin, China). The stock solution of fatty acids was prepared by dissolving individual standards in acetone and stored in a refrigerator. The working solution was further diluted prior to HPLC analysis.

**Serum FFAs profiling**

**Sample preparation**

An aliquot of 0.5 mL thawed serum sample was spiked with 10μL 2 mg/mL heptadecanoic acid as internal standard and 100μL 10% trichloroacetic acid, and then extracted with 2mL n-hexane three times by vortex-mixing for 2 min and centrifugated for 10 min at 12,000 rpm. The supernatant fluid was extracted and evaporated to dryness under nitrogen gas. The product was dissolved to 250μL by methanol and filtered through 0.22μm membrane prior to UPLC-QTOF analysis.

**Chromatographic condition**

Serum metabolite profiling was performed with UPLC-MS. Chromatography was carried out with Zorbax Aq-C18 column (150mm×3mm, i.d., 1.8μm, U.S. Agilent Company); the binary mobile phase was composed of phase A (0.2% methanoic acid in water) and phase B (acetonitrile).

The gradient for the serum sample was: 0-20min, 50-98% B. The proportion of phase B was kept at 100% level for 3min and then returned to 50% in 1min, and the column was allowed to re-equilibrate for 5min before the next injection. The flow rate was 0.6 mL/min, and 5μL was injected into the column. The column temperature was maintained at 40ºC.

**Q-TOF/MS conditions**

For MS, we used the ESI ion source, and the negative ion full scan mode (instrument mode 1700 m/z) was selected with maximum dynamic range. Nitrogen was used as the dry gas, the cone gas flow was maintained at 10.0 L/min, the desolvation temperature was set at 325ºC; atomization gas pressure at 50 psig; capillary voltage at 4000 V, and cone voltage at 65V, OCT 1 RF Vpp 750V; and fragment or voltage at 175 V. The TOF data were collected from 100-500 m/z.

**Method validation**

Using the analytical conditions mentioned above, fatty acid profiles from the ischemia and sham groups were obtained by the pre-column derivatization HPLC method. Additionally, the precision, recovery and line arranges of the conditions were also investigated. Relative standard deviation (RSD) ranged from 0.97% to 2.52% for inter-day assay, recoveries were in the range of 93.20 - 102.81%and the correlation coefficient (R^2^) of the calibration curves was higher than 0.991.

**Serum amino acid profiling**

**Sample preparation**

After 100 µL of the serum sample were taken, 800 µL pure water and 100 µL internal standard solution (positive valine 250 ng/ml) were added. Then, 200 µL of the supernatant fluid were collected, and 800 µL 85% methanol was added followed by vortexing for 5 min and standing for 1 h at 4 ºC. After centrifugation at 12,000 rpmfor 10 min, 200 µL of the supernatant fluid were taken, and clear liquid was obtained by filtration with a 0.22 microfiltration membrane. Finally, the samples were stored in liquid vialsfor online derivative HPLC - FLD testing method.

**Online derivatization methods**

Two adjacent benzene formaldehyde (OPA) for derivative reagent: measuring 1 mL of OPA reserve liquid (10 mg/ml), adding 1 mL borate buffer (pH=10.2), then 100 µL 3 - mercaptoethanol (3 - MPA), mixing all of these fluidsfollowed by filtration with a 0.22 µm membrane.

Derivatization process was performed automatically on Agilent 1200online automatic sampler, and the program was as follows: measuring 50 µL phosphate buffer solution (pH = 10.2), 5 µL serum samples, and 5 µL OPA derivative reagent, fully mixing 5 times, and waiting for the 1 min, at last inject the sample.

**HPLC analysis**

The chromatographic conditions used in this study were as follows: room temperature at 5 ºC; Hypersil C18 reversed phase chromatographic column (250 mm×4.6 mm, 5 µ m, DaLian Elite Analytical Instrument Co.,Ltd,China); mobile phase A liquid: 10 mmol/LNa_2_HPO_4_ - Na_2_B_4_O_7_PH = 7.95 buffer; mobile phase B for acetonitrile: methanol: water (volume ratio for 45:45:10). The elution condition we used was gradient elution: mobile phase B increased from 5% to 17.2% in 6 min, then increased from 17.2% to 52% from 6 min to 35 min; fluorescence detection wavelength of 338 nm (spectral bandwidth 10 nm), and reference wavelength of 390 nm (spectral bandwidth 10 nm).

**Method validation**

Relative standard deviation (RSD) ranged from 0.94% to 1.92% for inter-day assay, recoveries were in the range of 94.20-102.9%, and the correlation coefficient (R^2^) of the calibration curves was higher than 0.9443.

**Data processing**

Statistical analysis was performed using the SPSS software (Version 16.0, USA) and statistical significance was set at P < 0.05. Data were presented as means ± standard deviation for continuous variables; or as counts and percentages for categorical variables. The samples from the rats were analyzed grouped rather than individually. For cerebral infarction data we used *t* test, and for nerve function score data we used rank sum test.

**Multivariate statistical analysis**

Multivariate statistical analysis was performed by the software XCMS version 1.26.0 (<http://www.bioconductor.org/packages/2.8/bioc/html/xcms.html>) running under R version 2.13(http://www.r_project.org). Partial least squares-discriminant analysis (PLS-DA) model was then validated using cross validation and permutations. Routines of cross validation and permutation tests were in-house written and performed using Matlab 2011a.

The "centWave" method needs the chromatographic peak width range and the XCMS group command needs the bandwidth (half width at half maximum) of a Gaussian smoothing kernel. Therefore, plotChrom of XCMSwas first employed to observe the chromatogram. The optimized XCMS parameters were as follows: maximal tolerated m/z deviation in consecutive scans in parts per million(ppm) 15.0, peak width range (peakwidth) c (6,16),signal-to-noise cutoff(snthresh) 6.0,Gaussian smoothing-function width(bw) 2.0, minimum number of samples(minsamp) 1, width of overlapping m/z slice (mzwid) 0.01, and minimum fraction of samples in at least one of the sample groups (minfrac) 0.2. The parameters for ppm and snthresh were set according to the TOF MS instrument. After the m/z data of the metabolites were obtained by XCMS, further identification of the metabolites was carried out by Q-TOF/MS.

The metabolic profiles of disease cases and healthy controls were compared with the aim of identifying spectral features, and ultimately metabolites, which could discriminate the classes. Each spectrum was imported into XCMS software for retention time correction and filled in missing peak data. And then the data file was imported into Matlab software for Gios classify and PLS-DA.

**References**

Lin, T.N., He, Y.Y., Wu, G., Khan, M., Hsu, C.Y. (1993). Effect of brain edema on infarct volume in a focal cerebral ischemia model in rats.Stroke. 24,117-121.

Swanson,R.A., Morton, M.T., Tsao-Wu,G., et al.(1990). A semiautomated method for measuring brain infarct volume.J Cereb Blood Flow Metab. 10, 290-293.

Guo, Z.L., Zhu, Y., Su, X.T., et al. (2015). DanHong injection dose-dependently varies amino acid metabolites and metabolic pathways in the treatment of rats with cerebral ischemia. Acta Pharmacologica Sinica.36:748–757.

Xu, W.J., Zhang, L.X., Huang, Y.H., et al. (2012). Discrimination of type 2 diabetes mellitus corresponding to different traditional Chinese medicine syndromes based on plasma fatty acid profiles and chemometric methods. Journal of Ethnopharmacology, 143, 28: 463-468.

**2. Supplementary tables and figures**

Table S1 The known biological functions for 35 metabolites

| Metabolite | Fold | tR(min) | Exact mass | Relative peak area | Formula | changes | Report functions | References |
| --- | --- | --- | --- | --- | --- | --- | --- | --- |
| **Glutamic acid** | 1.38 | 7.661 | 147.0532 | 17.96268 | C_5_H_9_NO_4_ | ↑ | Glutamate is the most abundant excitatory neurotransmitter in the vertebrate nervous system.Because of its role in synaptic plasticity, glutamate is involved in cognitive functions such as learning and memory in the brain.In brain injury or disease, they can work in reverse, and excess glutamate can accumulate outside cells. Excessive glutamate release and impaired uptake occurs as part of the ischemic cascade and is associated with stroke. | [1-3] |
| **Asparagine** | 0.58 | 10.704 | 132.0535 | 4.35813 | C_4_H_8_N_2_O_3_ | ↓ | Asparagine is required for development and function of the brain. It also plays an important role in the synthesis of ammonia.Asparagine synthetase deficiency (ASD) is a newly identified neurometabolic disorder characterized by severe congenital microcephaly, severe global developmental delay, intractable seizure disorder, and spastic quadriplegia. | [4] |
| **Serine** | 0.56 | 11.709 | 105.0426 | 86.39092 | C_3_H_7_NO_3_ | ↓ | Serine and the products of serine metabolism are not only essential for cell proliferation, but are also necessary for specific functions in the central nervous system.Serine metabolism pathway might be associated with the incidence of cerebral ischemia. | [5,6] |
| **Glycine** | 0.88 | 14.557 | 75.032 | 77.44959 | C_2_H_5_NO_2_ | ↓ | Glycine is an inhibitory neurotransmitter in the central nervous system, especially in the spinal cord, brainstem, and retina. Glycine levels are effectively measured in plasma in both normal patients and individuals with inborn errors of glycine metabolism | [7] |
| **Threonine** | 1.23 | 18.055 | 174.1117 | 35.54111 | C_6_H_14_N_4_O_2_ | ↑ | Together with serine, threonine is one of two proteinogenic amino acids bearing an alcohol group.In humans, it is converted to α-ketobutyrate in a less common pathway via the enzyme serine dehydratase, and thereby enters the pathway leading to succinyl-CoA.The level of threonine is reported to be higher in patients with cirrhosis. | [8] |
| **Taurine** | 0.96 | 20.966 | 125.1457 | 55.17722 | C_2_H_7_NO_3_S | ↓ | Taurine is essential for cardiovascular function, and development and function of skeletal muscle, the retina, and the central nervous system.Taurine crosses the blood–brain barrier and has been implicated in a wide array of physiological phenomena including inhibitory neurotransmission, long-term potentiation in the striatum/hippocampus, membrane stabilization,feedback inhibition of neutrophil/macrophage respiratory burst, adipose tissue regulation and possible prevention of obesity, calcium homeostasis,recovery from osmotic shock, protection against glutamate excitotoxicity and prevention of epileptic seizures.Additionally, supplementation with taurine has been shown to prevent oxidative stress induced by exercise. | [9-18] |
| **Tyrosine** | 0.65 | 23.886 | 181.0739 | 13.36174 | C_9_H_11_NO_3_ | ↓ | Tyrosine improvements in cognitive and physical performance. | [19] |
| **Valine** | 1.64 | 26.357 | 117.079 | 116.29909 | C_5_H_11_NO_2_ | ↑ | NA |  |
| **Ornithine** | 0.63 | 31.178 | 132.0899 | 79.29524 | C_5_H_12_N2O_2_ | ↓ | Ornithine is a central part of the urea cycle. L-ornithine has an antifatigue effect in increasing the efficiency of energy consumption and promoting the excretion of ammonia. | [20,21 |
| **Leucine** | 1.18 | 31.858 | 131.0946 | 246.61391 | C_6_H_13_NO_2_ | ↑ | The level of leucine is reported to be higher in patients with cirrhosis. | [22] |
| **linolenic acid** | 1.21 | 13 | C18:3 | C_18_H_30_O_2_ | 278.22458 | ↑ | Gamma-linolenic acid induces apoptosis and lipid peroxidation , which is also thought to be an anti-inflammatory fatty acid | [23,24] |
| **Arachidic acid** | 0.89 | 14.565 | C20:0 | C_20_H_40_O_2_ | 312.3028 | ↓ | Arachidonic acid metabolism plays specific roles in the regulation of cerebral blood flow (CBF) , the modulation of vascular permeability, and the modulation of excitatory and inhibitory neurotransmitter release .Previous studies of arachidonic acid metabolism in experimental cerebral ischemia and reperfusion have shown that the accumulation of fatty acids, mainly arachidonic acid, due to the breakdown of structural membrane lipids, leads to significant variations of the concentrations of prostaglandins (PGs) in brain tissues . Once free, arachidonic acid undergoes both enzyme-independent and enzyme-mediated oxidative metabolism, resulting in the formation of number of biologically active metabolites which themselves contribute to pathological stroke outcomes . | [25-31] |
| **Palmitic acid** | 1.39 | 16.5 | C16:0 | C_16_H_32_O_2_ | 256.2402 | ↑ | According to the World Health Organization, evidence is "convincing" that consumption of palmitic acid increases risk of developing cardiovascular diseases. | [32] |
| **Metabolites** |  | **tR(min)** | **m/z** | **Actual Mass** | **Formula** |  |  |  |
| **Creatine** | 1.29 | 1.33 | 132.0716 | 131.0695 | C_4_H_9_N_3_O_2_ | ↑ | It has been shown that creatine supplementation has some positive effects on the central nervous system1; creatine-derived compounds may reproduce the neuroprotective effects of creatine while better crossing the neuronal plasma membrane and the blood-brain barrier. | [33,34] |
| **Uric acid** | 1.35 | 1.57 | 169.0268 | 168.0283 | C_5_H_4_NO_4_N_3_ | ↑ | An increased uric acid level was found to be associated with a decreased risk of poor outcomes among 3,231 acute stroke patients and silent brain infarction . | [35] |
| **Phytosphingosine** | 0.79 | 0.97 | 318.2006 | 317.293 | C_18_H_39_NO_3_ | ↓ | Prosopinine has an impact on the central and autonomic nervous systems. | [36] |
| **L-Valine** | 1.64 | 1.3 | 118.0863 | 117.079 | C_5_H_11_NO_2_ | ↑ | NA |  |
| **L-carnitine** | 1.4 | 1.28 | 162.112 | 161.1052 | C_7_H_15_NO_3_ | ↑ | Administration of acetyl-L-carnitine (ALCAR) attenuates neuronal damage, prevents apoptosis, and improves energy status in hypoxic stress through less understood mechanisms . Pre-treatment with chronic ALCAR significantly reduced the infarct size , and post-ischemic treatment with ALCAR did improve early clinical recovery and prevented significant weight loss in rat models of focal cerebral ischemia. | [37-38] |
| **L-acetylcarnitine** | 0.66 | 1.6 | 204.1218 | 203.1158 | C_9_H_17_NO_4_ | ↓ |  |  |
| **Glycerolphosphocholine(16:0)** | 1.38 | 18.37 | 497.3444 | 496.341 | C_8_H_20_NO_6_P | ↑ | Neurochemical monitoring has indicated that hypothermia decreases glycerol in the “tissue at risk” area of the infarct but not within the infarct core. | [39] |
| **13S-Hydroxyoctadecadienoic acid** | 0.99 | 21.95 | 297.2408 | 296.2351 | C18H32O3 | ↓ | 13-HODE may induce conformational changes in the vitronectin receptor to reduce its ability to recognize its adhesive ligands. The additional observations that 13-HODE levels in both human and animal vessel walls are inversely related with vessel wall adhesivity, | [40] |
| **Tyr Val / Val Tyr** | 1.28 | 19.7 | 281.1496 | 280.1423 | C_14_H_20_N_2_O_4_ | ↑ | NA |  |
| **6-hydroxysphingosine** | 1.93 | 18.7 | 316.2846 | 315.2773 | C_18_H_37_NO_3_ | ↑ | 6-hydroxysphingosine, a previously unknown long-chain base, is found in human skin. | [41] |
| **Prosopinine** | 0.72 | 15.83 | 288.2533 | 287.246 | C_16_H_33_NO_3_ | ↓ | Prosopinine is an alkaloid from Prosopis africana (Legumineous) and has some effects on the central and autonomic nervous systems. | [42-47] |
| **(3R)-3-isopropenyl-6-oxoheptanoic acid** | 0.89 | 19.7 | 185.1172 | 184.1099 | C_10_H_16_O_3_ | ↓ | NA |  |
| **cis-4-Decenoic acid** | 0.93 | 21.77 | 171.138 | 170.1307 | C_10_H_18_O_2_ | ↓ | Cis-4-decenoic acid is a characteristic metabolite in medium-chain acyl-CoA dehydrogenase deficiency.By measureing cis-4-decenoic acid in dried blood spots,one can diagnosis medium chain acyl CoA dehydrogenase deficiency. Na+, K+ ATPase activity is markedly reduced by cis-4-decenoic acid in synaptic plasma membranes from cerebral cortex of rats. | [47-53] |
| **Eicosapentaenoyl Serotonin** | 0.93 | 23.15 | 461.3163 | 460.309 | C_30_H_40_N_2_O_2_ | ↓ | NA |  |

[1] Meldrum, B. S. Glutamate as a neurotransmitter in the brain: Review of physiology and pathology. The Journal of nutrition. **130** (4S Suppl): 1007S–1015S (2000)

[2] McEntee, W. J.; & Crook, T. H. Glutamate: Its role in learning, memory, and the aging brain. Psychopharmacology. **111** (4): 391–401 (1993)

[3] Robert S. Biology and Human Behavior: The Neurological Origins of Individuality, 2nd edition. *The Teaching Company*. see pages 19 and 20 of Guide Book (2005)

[4] Alfadhel M, Alrifai MT, Trujillano D,et al.Asparagine Synthetase Deficiency: New Inborn Errors of Metabolism.*JIMD Rep*.**22**:11-6 (2015)

[5] Cynober LA. Plasma amino acid levels with a note on membrane transport: characteristics, regulation, and metabolic significance. *Nutrition*.**18**: 761-766(2002)

[6] Jung JY, Lee HS, Kang DG, Kim NS, Cha MH, Bang OS, et al. ^1^H-NMR- based metabolomics study of cerebral infarction. Stroke.**42**: 1282–1288(2011).

[7] Christie GR, Ford D, Howard A, Clark MA, Hirst BH. Glycine supply to human enterocytes mediated by high-affinity basolateral GLYT1.*Gastroenterology*.**120**: 439–448(2001)

[8] Dabos, K.J., Parkinson, J.A., Sadler, I.H.,et al.(1)H nuclear magnetic resonance spectroscopy-based metabonomic study in patients with cirrhosis and hepatic encephalopathy.*World J Hepatol*. **7**(12):1701-1707(2015)

[9] Huxtable, RJ. Physiological actions of taurine. *Physiol Rev*.**72** (1): 101–163(1992)

[10] Urquhart, N; Perry, TL; Tsuji, A; Tamai, I. Sodium- and chloride-dependent transport of taurine at the blood–brain barrier. *Advances in Experimental Medicine and Biology*.**403**: 385-391(1996)

[11] Salimäki, J, Scriba, G, Piepponen, TP, Rautolahti, N, Ahtee, L. The effects of systemically administered taurine and N-pivaloyltaurine on striatal extracellular dopamine and taurine in freely moving rats. *Naunyn-Schmiedeberg's Archives of Pharmacology* 368 (2): 134–41(2003)

[12] Olive, M.F. Interactions between taurine and ethanol in the central nervous system. *Amino Acids* 23 (4): 345–57(2002)

[13] Jr Dominy, J.; Thinschmidt, J.S.; Peris, J.; Dawson, R.; & Papke, R.L. Taurine-induced long-lasting potentiation in the rat hippocampus shows a partial dissociation from total hippocampal taurine content and independence from activation of known taurine transporters"". *Journal of Neurochemistry.* 89 (5): 1195–1205(2004)

[14] Birdsall, TC. Therapeutic applications of taurine. *Alternative Medicine Review.* 3 (2): 128–36(1998).

[15] Ide T, Kushiro M, Takahashi Y, Shinohara K, Cha S. mRNA expression of enzymes involved in taurine biosynthesis in rat adipose tissues. *Metabolism: Clinical and Experimental.* 51(9):1191-7(2002)

[16] Tsuboyama-Kasaoka, N; Shozawa, C; Sano, K; Kamei, Y; Kasaoka, S; Hosokawa, Y; Ezaki, O. Taurine (2-aminoethanesulfonic acid) deficiency creates a vicious circle promoting obesity. *Endocrinology.* 147 (7): 3276–84(2006)

[17] Foos, TM; Wu, JY. The role of taurine in the central nervous system and the modulation of intracellular calcium homeostasis. *Neurochemical Research .*27 (1–2): 21–6(2002)

[18] Stummer W, Betz AL, Shakui P, Keep RF. Blood–brain barrier taurine transport during osmotic stress and in focal cerebral ischemia. *Journal of Cerebral Blood Flow and Metabolism.* 15 (5): 852–859(1995)

[19] Leon R, Wu H, Jin Y, Wei J, Buddhala C, Prentice H, Wu JY. Protective function of taurine in glutamate-induced apoptosis in cultured neurons. *Journal of Neuroscience Research* 87: 1185–1194(2008)

[20] El Idrissi A, Messing J, Scalia J, Trenkner E; Messing; Scalia; Trenkner. Prevention of epileptic seizures by taurine. *Advances in Experimental Medicine and Biology* 526: 515–25(2003)

[21]Mahoney CR, Castellani J, Kramer FM, Young A, Lieberman HR. Tyrosine supplementation mitigates working memory decrements during cold exposure. *Physiology and Behavior.* IN PRESS (4): 575-82(2007)

[22] Sugino, T; Shirai, T; Kajimoto, Y; Kajimoto, O. L-ornithine supplementation attenuates physical fatigue in healthy volunteers by modulating lipid and amino acid metabolism. *Nutrition research* 28 (11): 738-43(2008)

[23] Demura, S; Yamada, T; Yamaji, S; Komatsu, M; Morishita, K. The effect of L-ornithine hydrochloride ingestion on performance during incremental exhaustive ergometer bicycle exercise and ammonia metabolism during and after exercise. *European journal of clinical nutrition* 64 (10): 1166–71(2010)

[24] Dabos KJ1, Parkinson JA1, Sadler IH1,et al. H nuclear magnetic resonance spectroscopy-based metabonomic study in patients with cirrhosis and hepatic encephalopathy.*World J Hepatol*. 28;7(12):1701-1707 (2015)

[25] Ge, H. et al. Gamma-linolenic acid induces apoptosis and lipid peroxidation in human chronic myelogenous leukemia K562 cells.*Cell Biol Int* (2009)

[26] Kapoor, R., Huang, Y.S. Gamma linolenic acid: an antiinflammatory omega-6 fatty acid.*Curr Pharm Biotechnol*.7:531-534(2006)

[27]Wick, G., Perschinka, H. Millonig,G. Atherosclerosis as an auto-immune disease: an update. *Trends Immnol*. 22:665-669(2001)

[28] Leslie, J.B. Watkins, W.D. Eicosanoids in the central nervous system. *J Neurosurg* .63:659-668 (1985)

[29] Kimura, H., Okamoto, K., Sakai,Y. Modulatory effects of prostaglandin D2, E2, F2a on the postsynaptic actions of inhibitory and excitatory amino acids in cerebellar Purkinje cell dendrites "in vitro." *Brain Res*.330:235-244(1997)

[30] Pickard, J.D. Role of prostaglandins and arachidonic acid derivatives in the coupling of cerebral blood flow to cerebral *metabolism*.(1981)

[31] Kempski,O. et al. Postischemic production of eicosanoids in gerbil brain. *Stroke.*18:111-119(1987)

[32] Shoami, E., Rosenthal, J. Lavy, S. The effect of incomplete cerebral ischemia on prostaglandin levels in rat brain. *Stroke*.13:494-499(1982)

[33] Gaudet,R.J. Levine,L.Effect of unilateral common carotid artery occlusion on levels of prostaglandins D2, F2 and 6-keto-prostaglandin Fla in gerbil brain. *Stroke*, 11:648-652(1980)

[34] Rink C, Khanna S. Significance of brain tissue oxygenation and the arachidonicacid cascade in stroke. *Antioxid Redox Signal*. May 15;14(10):1889-903(2011)

[35] Diet, Nutrition and the Prevention of Chronic Diseases, WHO Technical Report Series 916, Report of a Joint WHO/FAO Expert Consultation, *World Health Organization*, Geneva. 88 (Table 10) (2003)

[36] Perasso,L., Spallarossa,P., Gandolfo,C. Therapeutic use of creatine in brain or heart ischemia: available data and future perspectives. *Med Res Rev*.33:336-363(2013)

[37] Klein, A.M. Ferrante, R.J. The neuroprotective role of creatine.*Subcell Biochem*.46:205-243(2007)

[38] Zhang, X.L. et al.Association between serum uric acid and short-term clinical outcome among patients with acute *stroke*.15;14(10):1877-1903 (2003).

[39]Heo, S.H., Lee, S.H. High levels of serum uric acid are associated with silent brain infarction.*J Neurol Sci*. 297:6-10(2010)

[40] Brouns, R. et al. Decrease in uric acid in acute ischemic stroke correlates with stroke severity, evolution and outcome. *Clin Chem Lab Med*. 48:383-390(2010)

[41] Bourrinet, P., Quevauviller, A. Prosopinine, an alkaloid from Prosopis africana (Legumineous). Its effects on the central and autonomic nervous systems. *C R Seances Soc Biol Fil*. 162:1138-1140(1968)

[42] Jalal,F.Y., Böhlke,M. Maher,T.J. Acetyl-L-carnitine reduces the infarct size and striatal glutamate outflow following focal cerebral ischemia in rats. *Ann N Y Acad Sci*.1199:95-104(2010)

[43] Lolic, M.M., Fiskum, G. Rosenthal, R.E. Neuroprotective effects of acetyl-L-carnitine after stroke in rats. *Ann Emerg Med.*,29:758-765 (1997)

[44] Christian, B., Wolf-Rudiger, S., Dimitrios, G. Effects of Hypothermia on Excitatory AminoAcids and Metabolism in Stroke Patients : A Microdialysis Study.*Stroke*. 33:519-524(2002)

[45] Buchanan, M.R., Bertomeu, M.C., Brister, S.J., et al. 13-Hydroxyoctadecadienoic acid (13-HODE) metabolism and endothelial cell adhesion molecule expression: effect on platelet vessel wall adhesion.*Wien Klin Wochenschr.* 103(14):416-21(1991)

[46] Stewart, M.E., Downing, D.T. Free sphingosines of human skin include 6-hydroxysphingosine and unusually long-chain dihydrosphingosines.*J Invest Dermatol*. 105(4):613-8(1995)

[47]Duran, M., Bruinvis, L., Ketting, D., et al. Cis-4-decenoic acid in plasma: a characteristic metabolite in medium-chain acyl-CoA dehydrogenase deficiency. *Clin Chem.* 34(3):548-51(1988).

[48] Heales, S.J., Leonard, J.V.Diagnosis of medium chain acyl CoA dehydrogenase deficiency by measurement of cis-4-decenoic acid in dried blood spots. *Clin Chim Acta.* 31;209(1-2):61-66 (1992)

[49]Assis, D.R., Maria, R.C., Ferreira, G.C., et al. Na+, K+ ATPase activity is markedly reduced by cis-4-decenoic acid in synaptic plasma membranes from cerebral cortex of rats. *Exp Neurol*. 197(1):143-149 (2006)

[50] Barber,M.N. et al. Plasma lysophosphatidylcholine levels are reduced in obesity and type 2 diabetes. *PLoS One*.7:e41456 (2012)

[51] Park, S., Kim, J.A., Choi, S. Suh, S.H. Superoxide is a potential culprit of caspase-3 dependent endothelial cell death induced by lysophosphatidylcholine.*J Physiol Pharmacol*.61:375-381(2010)

[52] Song, J. et al. Luteolin inhibits lysophosphatidylcholine-induced apoptosis in endothelial cells by a calcium/mitocondrion/ caspases-dependent pathway. *Planta Med.*76:433-438 (2010)

[53] Gonçalves, I. et al. Evidence supporting a key role of Lp-PLA2-generated lysophosphatidylcholine in human atherosclerotic plaque inflammation.*Arterioscler Thromb Vasc Biol*.32:1505-1512 (2012)

Table S2 Compound name, synonyms, formula, 2D structure, molecular weight, CAS number and smiles string for the nodes in compound network map

| ID | Name | Formula | Mean Weight |
| --- | --- | --- | --- |
| C00002 | ATP | C_10_H_16_N_5_O_13_P_3_ | 507.1810 |
| C00011 | CO_2_ | CO_2_ | 44.0095 |
| C00014 | NH_3_ | H_3_N | 17.0305 |
| C00022 | Pyruvate | C_3_H_4_O_3_ | 88.0621 |
| C00025 | L-Glutamate | C_5_H_9_NO_4_ | 147.1293 |
| C00026 | 2-Oxoglutarate | C_5_H_6_O_5_ | 146.0981 |
| C00036 | Oxaloacetate | C_4_H_4_O_5_ | 132.0716 |
| C00037 | Glycine | C_2_H_5_NO_2_ | 75.0666 |
| C00041 | L-Alanine | C_3_H_7_NO_2_ | 89.0932 |
| C00048 | Glyoxylate | C_2_H_2_O_3_ | 74.0355 |
| C00049 | L-Aspartate | C_4_H_7_NO_4_ | 133.1027 |
| C00051 | Glutathione | C_10_H_17_N_3_O_6_S | 307.3235 |
| C00062 | L-Arginine | C_6_H_14_N_4_O_2_ | 174.2010 |
| C00064 | L-Glutamine | C_5_H_10_N_2_O_3_ | 146.1445 |
| C00065 | L-Serine | C_3_H_7_NO_3_ | 105.0926 |
| C00079 | L- Phenylalanine | C_9_H_11_NO_2_ | 165.1891 |
| C00082 | L-Tyrosine | C_9_H_11_NO_3_ | 181.1885 |
| C00091 | Succinyl-CoA | C_25_H_40_N_7_O_19_P_3_S | 867.6069 |
| C00097 | L-Cysteine | C_3_H_7_NO_2_S | 121.1582 |
| C00101 | Tetrahydro-  folate | C_19_H_23_N_7_O_6_ | 445.4292 |
| C00114 | Choline | C_5_H_14_NO | 104.1708 |
| C00123 | L-Leucine | C_6_H_13_NO_2_ | 131.1729 |
| C00141 | 3-Methyl-2- oxobutanoic acid | C_5_H_8_O_3_ | 116.1152 |
| C00143 | 5,10-Methylenetetrahydrofolate | C_20_H_23_N_7_O_6_ | 457.4399 |
| C00152 | L-Asparagine | C_4_H_8_N_2_O_3_ | 132.1179 |
| C00154 | Palmitoyl-CoA | C_37_H_66_N_7_O_17_P_3_S | 1005.9429 |
| C00155 | L- Homocysteine | C_4_H_9_NO_2_S | 135.1848 |
| C00157 | Phosphatidylcholine | C_10_H_18_NO_8_PR_2_ | - |
| C00168 | Hydroxypyruvate | C_3_H_4_O_4_ | 104.0615 |
| C00183 | L-Valine | C_5_H_11_NO_2_ | 117.1463 |
| C00188 | L-Threonine | C_4_H_9_NO_3_ | 119.1192 |
| C00189 | Ethanolamine | C_2_H_7_NO | 61.0831 |
| C00213 | Sarcosine | C_3_H_7_NO_2_ | 89.0932 |
| C00219 | Arachidonate | C_20_H_32_O_2_ | 304.4669 |
| C00233 | 4-Methyl-2- oxopentanoate | C_6_H_10_O_3_ | 130.1418 |
| C00245 | Taurine | C_2_H_7_NO_3_S | 125.1469 |
| C00249 | Hexadecanoic acid | C_16_H_32_O_2_ | 256.4241 |
| C00268 | Dihydro- biopterin | C_9_H_13_N_5_O_3_ | 239.2312 |
| C00272 | Tetrahydro- biopterin | C_9_H_15_N_5_O_3_ | 241.2471 |
| C00334 | 4-Amino butanoate | C_4_H_9_NO_2_ | 103.1198 |
| C00350 | Phosphatidylethanolamine | C_7_H_12_NO_8_PR_2_ | - |
| C00355 | 3,4-Dihydroxy-L-  phenylalanine | C_9_H_11_NO_4_ | 197.1879 |
| C00366 | Urate | C_5_H_4_N_4_O_3_ | 168.1103 |
| C00370 | Sterol | C_17_H_28_O | 248.4036 |
| C00385 | Xanthine | C_5_H_4_N_4_O_2_ | 152.1109 |
| C00416 | Phosphatidate | C_5_H_7_O_8_PR_2_ | - |
| C00430 | 5-Aminolevulinate | C_5_H_9_NO_3_ | 131.1299 |
| C00439 | N-Formimino-L-glutamate | C_6_H_10_N_2_O_4_ | 174.1546 |
| C00483 | Tyramine | C_8_H_11_NO | 137.1790 |
| C00487 | Carnitine | C_7_H_16_NO_3_ | 162.2068 |
| C00506 | L-Cysteate | C_3_H_7_NO_5_S | 169.1564 |
| C00542 | Cystathionine | C_7_H_14_N_2_O_4_S | 222.2621 |
| C00581 | Guanidinoacetate | C_3_H_7_N_3_O_2_ | 117.1066 |
| C00624 | N-Acetyl- L-glutamate | C_7_H_11_NO_5_ | 189.1659 |
| C00630 | 2- Methylpropanoyl-CoA | C_25_H_42_N_7_O_17_P_3_S | 837.6240 |
| C00664 | 5-Formiminotetrahydrofolate | C_20_H_24_N_8_O_6_ | 472.4546 |
| C00669 | gamma-  L-Glutamyl- L-cysteine | C_8_H_14_N_2_O_5_S | 250.2722 |
| C00670 | sn-glycero-3- Phosphocholine | C_8_H_21_NO_6_P | 258.2292 |
| C00708 | Iodide | I | 127.9124 |
| C00787 | tRNA(Tyr) | C_15_H_21_N_5_O_10_PR(C_5_H_8_O_6_PR)n | - |
| C00822 | Dopaquinone | C_9_H_9_NO_4_ | 195.1721 |
| C01005 | O-Phospho-L- serine | C_3_H_8_NO_6_P | 185.0725 |
| C01033 | 2-Methylbutanoyl-CoA | C_26_H_44_N_7_O_17_P_3_S | 851.6505 |
| C01149 | 4-Trimethylammoniobutanal | C_7_H_16_NO | 130.2080 |
| C01165 | L-Glutamate 5-semialdehyde | C_5_H_9_NO_3_ | 131.1299 |
| C01179 | 3-(4-Hydroxyphenyl)pyruvate | C_9_H_8_O_4_ | 180.1574 |
| C01242 | S-Amino methyldihydro lipoylprotein | C_9_H_18_NOS_2_R | - |
| C01259 | 3-Hydroxy-N6,N6,N6-trimethyl-L-lysine | C_9_H_21_N_2_O_3_ | 205.2746 |
| C01419 | Cys-Gly | C_5_H_10_N_2_O_3_S | 178.2095 |
| C01595 | Linoleate | C_18_H_32_O_2_ | 280.4455 |
| C01637 | tRNA(Asn) | C_10_H_17_O_10_PR_2_  (C_5_H_8_O_6_PR)n | - |
| C01641 | tRNA(Glu) | C_15_H_21_N_5_O_10_PR(C_5_H_8_O_6_PR)n | - |
| C01642 | tRNA(Gly) | C_10_H_17_O_10_PR_2_  (C_5_H_8_O_6_PR)n | - |
| C01645 | tRNA(Leu) | C_15_H_21_N_5_O_10_PR(C_5_H_8_O_6_PR)n | - |
| C01650 | tRNA(Ser) | C_10_H_17_O_10_PR_2_  (C_5_H_8_O_6_PR)n | - |
| C01651 | tRNA(Thr) | C_10_H_17_O_10_PR_2_  (C_5_H_8_O_6_PR)n | - |
| C01653 | tRNA(Val) | C_15_H_21_N_5_O_10_PR(C_5_H_8_O_6_PR)n | - |
| C01794 | Choloyl-CoA | C_45_H_74_N_7_O_20_P_3_S | 1158.0902 |
| C01879 | 5-Oxoproline | C_5_H_7_NO_3_ | 129.1140 |
| C01921 | Glycocholate | C_26_H_43_NO_6_ | 465.6227 |
| C01958 | Steryl ester | C_18_H_27_O_2_R | - |
| C02047 | L-Leucyl-tRNA | C_21_H_32_N_6_O_11_PR(C_5_H_8_O_6_PR)n | - |
| C02050 | Linoleoyl-CoA | C_39_H_66_N_7_O_17_P_3_S | 1029.9643 |
| C02051 | Lipoylprotein | C_12_H_18_N_2_O_4_S_2_R_2_(C_2_H_2_NOR)n | - |
| C02218 | 2- Aminoacrylate | C_3_H_5_NO_2_ | 87.0773 |
| C02249 | Arachidonyl- CoA | C_41_H_66_N_7_O_17_P_3_S | 1053.9857 |
| C02291 | L-Cystathionine | C_7_H_14_N_2_O_4_S | 222.2621 |
| C02320 | R-S- Glutathione | C_10_H_16_N_3_O_6_SR | - |
| C02412 | Glycyl-tRNA (Gly) | C_12_H_20_NO_11_PR_2_(C_5_H_8_O_6_PR)n | - |
| C02515 | 3-Iodo-L- tyrosine | C_9_H_10_INO_3_ | 307.0851 |
| C02553 | L-Seryl-tRNA (Ser) | C_13_H_22_NO_12_PR_2_(C_5_H_8_O_6_PR)n | - |
| C02554 | L-Valyl-tRNA (Val) | C_20_H_30_N_6_O_11_PR(C_5_H_8_O_6_PR)n | - |
| C02571 | O- Acetylcarnitine | C_9_H_18_NO_4_ | 204.2435 |
| C02592 | Taurolithocholate | C_26_H_45_NO_5_S | 483.7042 |
| C02737 | Phosphatidylserine | C_8_H_12_NO_10_PR_2_ | - |
| C02839 | L-Tyrosyl- tRNA(Tyr) | C_24_H_30_N_6_O_12_PR(C_5_H_8_O_6_PR)n | - |
| C02934 | 3-Dehydrosphinganine | C_18_H_37_NO_2_ | 299.4919 |
| C02939 | 3-Methylbutanoyl-CoA | C_26_H_44_N_7_O_17_P_3_S | 851.6505 |
| C02987 | L-Glutamyl- tRNA(Glu) | C_20_H_28_N_6_O_13_PR(C_5_H_8_O_6_PR)n | - |
| C02992 | L-Threonyl- tRNA(Thr) | C_14_H_24_NO_12_PR_2_(C_5_H_8_O_6_PR)n | - |
| C03069 | 3-Methylcrotonyl-CoA | C_26_H_42_N_7_O_17_P_3_S | 849.6347 |
| C03287 | L-Glutamyl 5-phosphate | C_5_H_10_NO_7_P | 227.1092 |
| C03402 | L-Asparaginyl-tRNA(Asn) | C_14_H_23_N_2_O_12_PR_2_(C_5_H_8_O_6_PR)n | - |
| C03508 | L-2-Amino-3- oxobutanoic acid | C_4_H_7_NO_3_ | 117.1033 |
| C03541 | Tetrahydrofolyl-Glu(n) | C_24_H_30_N_8_O_9_(C_5_H_7_NO_3_)n | 574.5432 |
| C03680 | 4-Imidazolone-5-propanoate | C_6_H_8_N_2_O_3_ | 156.1393 |
| C03912 | (S)-1-Pyrroline-5-carboxylate | C_5_H_7_NO_2_ | 113.1146 |
| C03990 | 3alpha-Hydroxy-5beta-cholanate | C_24_H_40_O_3_ | 376.5726 |
| C04230 | 1-Acyl-sn-glycero-3-phosphocholine | C_9_H_20_NO_7_PR | - |
| C04233 | 2-Acyl-sn-glycero-3-phosphocholine | C_9_H_20_NO_7_PR | - |
| C04717 | 13(S)-HPODE | null | 312.4443 |
| C05122 | Taurocholate | C_26_H_45_NO_7_S | 515.703 |
| C05337 | Chenodeoxycholoyl-CoA | C_45_H_74_N_7_O_19_P_3_S | 1142.0908 |
| C05356 | 5(S)-HPETE | C_20_H_32_O_4_ | 336.4657 |
| C05463 | Taurodeoxycholate | C_26_H_45_NO_6_S | 499.7036 |
| C05465 | Taurochenodeoxycholate | C_26_H_45_NO_6_S | 499.7036 |
| C05519 | L-Allothreonine | C_4_H_9_NO_3_ | 119.1192 |
| C05520 | 2-Amino-3-oxoadipate | C_6_H_9_NO_5_ | 175.1394 |
| C05726 | R-S-Alanine | C_3_H_6_NO_2_SR | - |
| C05729 | R-S-Alanylglycine | C_5_H_9_N_2_O_3_SR | - |
| C05764 | Hexadecanoyl-"acp" | C_16_H_31_OSR | - |
| C05844 | 5-Glutamyl-taurine | C_7_H_14_N_2_O_6_S | 254.2609 |
| C05951 | Leukotriene D4 | C_25_H_40_N_2_O_6_S | 496.6599 |
| C05952 | Leukotriene E_4_ | C_23_H_37_NO_5_S | 439.6086 |
| C05956 | Prostaglandin G_2_ | C_20_H_32_O_6_ | 368.4645 |
| C05965 | 12(S)-HPETE | C_20_H_32_O_4_ | 336.4657 |
| C05966 | 15(S)-HPETE | C_20_H_32_O_4_ | 336.4657 |
| C06112 | L-Glutamyl-tRNA(Gln) | C_20_H_28_N_6_O_13_PR(C_5_H_8_O_6_PR)n | - |
| C06426 | (6Z,9Z,12Z)-Octadecatrienoic acid | C_18_H_30_O_2_ | 278.4296 |
| C06427 | (9Z,12Z,15Z)-Octadecatrienoic acid | C_18_H_30_O_2_ | 278.4296 |
| C09332 | Tetrahydrofolyl-"Glu"(2) | C_24_H_30_N_8_O_9_ | 574.5432 |
| C11695 | Anandamide | C_22_H_37_NO_2_ | 347.5347 |
| C11821 | 5-Hydroxyisourate | C_5_H_4_N_4_O_4_ | 184.1097 |
| C12144 | Phytosphingosine | C_18_H_39_NO_3_ | 317.5072 |
| C12145 | Phytoceramide | C_19_H_38_NO_4_R | - |
| C14748 | 20-HETE | C_20_H_32_O_3_ | 320.4663 |
| C14749 | 19(S)-HETE | C_20_H_32_O_3_ | 320.4663 |
| C14768 | 5,6-EET | C_20_H_32_O_3_ | 320.4663 |
| C14769 | 8,9-EET | C_20_H_32_O_3_ | 320.4663 |
| C14770 | 11,12-EET | C_20_H_32_O_3_ | 320.4663 |
| C14771 | 14,15-EET | C_20_H_32_O_3_ | 320.4663 |
| C14778 | 16(R)-HETE | C_20_H_32_O_3_ | 320.4663 |
| C14779 | 9(S)-HETE | C_20_H_32_O_3_ | 320.4663 |
| C14825 | 9(10)-EpOME | C_18_H_32_O_3_ | 296.4449 |
| C14826 | 12(13)-EpOME | C_18_H_32_O_3_ | 296.4449 |
| C14827 | 9(S)-HPODE | C_18_H_32_O_4_ | 312.4443 |
| CE0074 | alloxan | C_4_H_2_N_2_O_4_ | - |
| CE1450 | 11-hydroxyeicosatetraenoate | C_20_H_31_O_3_ | - |
| CE1556 | N-acetyl-L-asparagine | C_6_H_9_N_2_O_4_ | - |
| CE1589 | deoxycholoyl-CoA | C_45_H_70_N_7_O_19_P_3_S | - |
| CE1951 | tetrahydrobiopterin-4a-carbinolamine | C_9_H_16_N_5_O_4_ | - |
| CE2020 | N-arachidonoylglycine | C_22_H_34_NO_3_ | - |
| CE2026 | 3-methylcrotonoylglycine | C_7_H_10_NO_3_ | - |
| CE2565 | 15(R)-Hydroxy-(5Z,8Z,11Z,13E)-eicosatetraenoate | C_20_H_31_O_3_ | - |
| CE4809 | alpha-linolenoyl-CoA | C_39_H_60_N_7_O_17_P_3_S | - |
| CE4968 | isovalerylglycine | C_7_H_12_NO_3_ | - |
| CE4969 | isobutyrylglycine | C_6_H_10_NO_3_ | - |
| CE4970 | 2-methylbutyrylglycine | C_7_H_12_NO_3_ | - |
| CE4994 | 10,11-dihydro-12R-hydroxy-leukotriene D_4_ | C_25_H_41_N_2_O_7_S | - |
| CE4995 | 10,11-dihydro-12R-hydroxy-leukotriene E_4_ | C_23_H_38_NO_6_S | - |
| CE5560 | glycolithocholate | C_26_H_42_NO_4_ | - |
| CE5705 | 8-hydroxyeicosatetraenoate | C_20_H_31_O_3_ | - |
| CE7228 | 13-HETE | C_20_H_31_O_3_ | - |
| CE7231 | 7-HETE | C_20_H_31_O_3_ | - |
| CE7234 | 10-HETE | C_20_H_31_O_3_ | - |
| CE7243 | 18-HETE | C_20_H_31_O_3_ | - |
| CE7244 | 17-HETE | C_20_H_31_O_3_ | - |

Table S3 Comparing the pathways and the modular nodes

|  | Name | Identical nodes | Unique for the pathway | Unique for the module |
| --- | --- | --- | --- | --- |
| 1 | Glycine module and glycine, serine, alanine, threonine metabolism pathway | CO2  NH3  Glycine  Glyoxylate  L-Arginine  Sarcosine  Guanidinoacetate  2-Methylpropanoyl-CoA  2-Methylbutanoyl-CoA  S-Aminomethyldihydrolipoylprotein  tRNA(Gly)  Lipoylprotein  Glycyl-tRNA(Gly)  3-Methylbutanoyl-CoA  3-Methylcrotonyl-CoA  L-2-Amino-3-oxobutanoic acid  L-Allothreonine  N-arachidonoylglycine  3-methylcrotonoylglycine  isovalerylglycine  isobutyrylglycine  2-methylbutyrylglycine | Pyruvate  L-Serine  Hydroxypyruvate  L-Threonine  O-Phospho-L-serine  tRNA(Ser)  tRNA(Thr)  Arachidonyl-CoA  L-Seryl-tRNA(Ser)  L-Threonyl-tRNA(Thr) | Succinyl-CoA  5,10-Methylenetetrahydrofolate  5-Aminolevulinate  4-Trimethylammoniobutanal  3-Hydroxy-N6,N6,N6-trimethyl-L-lysine  Glycocholate  2-Amino-3-oxoadipate  R-S-Alanine  Leukotriene D4  Leukotriene E4  10,11-dihydro-12R-hydroxy-leukotriene D4  10,11-dihydro-12R-hydroxy-leukotriene E4  glycolithocholate  somatostatin  Somatostatin fragment 3-14 |
| 2 | L-glutamate module and  urea cycle and metabolism of arginine, proline, glutamate, aspartate and asparagine pathways | L-Glutamate  2-Oxoglutarate  Oxaloacetate  L-Alanine  L-Aspartate  Glutathione  L-Glutamine  4-Aminobutanoate  N-Acetyl-L-glutamate  gamma-L-Glutamyl-L-cysteine  L-Glutamate 5-semialdehyde  Cys-Gly  tRNA(Glu)  5-Oxoproline  R-S-Glutathione  L-Glutamyl-tRNA(Glu)  L-Glutamyl 5-phosphate  (S)-1-Pyrroline-5-carboxylate  R-S-Alanylglycine  L-Glutamyl-tRNA(Gln) | Pyruvate  Glycine  L-Cysteine  L-Asparagine  Carnitine  tRNA(Asn)  O-Acetylcarnitine  L-Asparaginyl-tRNA(Asn)  R-S-Alanine  N-acetyl-L-asparagine | ATP  Tetrahydrofolate  2-Methyl-3-oxopropanoate  N-Formimino-L-glutamate  5-Formiminotetrahydrofolate  (R)-3-Amino-2-methylpropanoate  Tetrahydrofolyl-[Glu](n)  4-Imidazolone-5-propanoate  Tetrahydrofolyl-[Glu](2) |
| 3 | Arachidonate module and  arachidonic acid pathway | Arachidonate  12(S)-HPETE  15(S)-HPETE  20-HETE  19(S)-HETE  5,6-EET  8,9-EET  11,12-EET  14,15-EET  16(R)-HETE  9(S)-HETE  11-hydroxyeicosatetraenoate  15(R)-Hydroxy-(5Z,8Z,11Z,13E)-eicosatetraenoate  8-hydroxyeicosatetraenoate  13-HETE  7-HETE  10-HETE  18-HETE  17-HETE | Phosphatidylcholine | Ethanolamine  Arachidonyl-CoA  5(S)-HPETE  Prostaglandin G2  Anandamide |
| 4 | L-serine module and urea cycle and metabolism of arginine, proline, glutamate, aspartate and asparagine pathways | Pyruvate  L-Cysteine | L-Glutamate  2-Oxoglutarate  Oxaloacetate  Glycine  L-Alanine  L-Aspartate  Glutathione  L-Glutamine  L-Asparagine  4-Aminobutanoate  Carnitine  N-Acetyl-L-glutamate  gamma-L-Glutamyl-L-cysteine  L-Glutamate 5-semialdehyde  Cys-Gly  tRNA(Asn)  tRNA(Glu)  5-Oxoproline  R-S-Glutathione  O-Acetylcarnitine  L-Glutamyl-tRNA(Glu)  L-Glutamyl 5-phosphate  L-Asparaginyl-tRNA(Asn)  (S)-1-Pyrroline-5-carboxylate  R-S-Alanine  R-S-Alanylglycine  L-Glutamyl-tRNA(Gln)  N-acetyl-L-asparagine | L-Serine  Choline  L-Homocysteine  Hydroxypyruvate  Phosphatidylethanolamine  Phosphatidate  Cystathionine  O-Phospho-L-serine  tRNA(Ser)  2-Aminoacrylate  L-Cystathionine  L-Seryl-tRNA(Ser)  Phosphatidylserine  3-Dehydrosphinganine |
| 5 | Tyrosine module and  tyrosine metabolism pathway | L-Tyrosine  Dihydrobiopterin  Tetrahydrobiopterin  3,4-Dihydroxy-L-phenylalanine  Tyramine  Iodide  tRNA(Tyr)  Dopaquinone  3-(4-Hydroxyphenyl)pyruvate  3-Iodo-L-tyrosine  L-Tyrosyl-tRNA(Tyr) | L-Phenylalanine | alpha-Tubulin  Detyrosinated alpha-tubulin  tetrahydrobiopterin-4a-carbinolamine |
| 6 | L-leucine module and  valine,leucine and isoleucine metabolism pathways | tRNA(Leu)  L-Leucyl-tRNA  L-Leucine  4-Methyl-2-oxopentanoate | tRNA(Val)  L-Valine  L-Valyl-tRNA(Val)  3-Methyl-2-oxobutanoic acid | L-Phenylalanine  kinetensin  kinetensin 1-3  kinetensin 1-7  kinetensin 1-8  kinetensin 4-7  kinetensin 4-8  neuromedin N  neuromedin N (1-4) |
| 7 | Taurine module and bile acid biosynthesis | Taurine  Choloyl-CoA  Taurolithocholate  3alpha-Hydroxy-5beta-cholanate  Taurocholate  Chenodeoxycholoyl-CoA  Taurodeoxycholate  Taurochenodeoxycholate  deoxycholoyl-CoA | Glycine  Glycocholate  glycolithocholate | L-Cysteate  5-Glutamyl-taurine |
| 8 | Linoleate module and  linoleate metabolism pathway | Phosphatidylcholine  Linoleate  13(S)-HPODE  (6Z,9Z,12Z)-Octadecatrienoic acid  9(10)-EpOME  12(13)-EpOME  9(S)-HPODE | 1-Acyl-sn-glycero-3-phosphocholine | Linoleoyl-CoA |

Table S4 Stroke-related pathways that can be verified from literature

| Pathway | Abbrev. | Literature |
| --- | --- | --- |
| Glycine, serine and threonine metabolism | GST metab | Stanton,P.K.,Potter,P.E.,Aguilar J.,.et al. (2009).Neuroprotection by a novel NMDAR functional glycine site partial agonist, GLYX-13.Neuroreport.,13,193-197. |
| Valine, leucine and isoleucine biosynthesis | VLI biosyn | Kimberly,W.T.,Wang,Y.,Pham,L.,et al.(2013).Metabolite profiling identifies a branched chain amino acid signature in acute cardioembolic stroke.Stroke.44,1389-1395. |
| Taurine and hypotaurine metabolism | TaH metab | Malin,H.S.,Mattias T., Stephen,G.W.,et al.(2008).Stimulated efflux of amino acids and glutathione from cultured hippocampal slices by omission of extracellular calcium: likely involvement of connexin hemichannels. The Journal of Biological Chemistry. 283,10347-10356. |
| D-Glutamine and D-glutamate metabolism | DGG metab | Hage,M.E.,Baverel,G.,Conjard,D.A.,et al.(2013).Effect of Glucose on Glutamine Metabolism in Rat Brain Slices: a Cellular Metabolomic Study with <sup>13</sup>C NMR. Neuroscience.248,243–251. |
| Alanine, aspartate and glutamate metabolism | AAG metab | Helene,B., Jørgen,D., Arne,S., Nils,H.D.(1984)Elevation of the Extracellular Concentrations of Glutamate and Aspartate in Rat Hippocampus During Transient Cerebral Ischemia Monitored by Intracerebral Microdialysis. Journal of Neurochemistry.43,1369–1374. |
| Glutathione metabolism | Glu metab | Carmina,B.,Wei,L.,Gloria,C.,et al.(2013).Inhibition of glutathione synthesis in brain endothelial cells lengthens S-phase transit time in the cell cycle: Implications for proliferation in recovery from oxidative stress and endothelial cell damage.Redox Biology.1,131–139. |
|  |  | Natacha,T.,Xavier,R., Nadia,W.,et al.(2012).Blood glutathione S-transferase-π as a time indicator of stroke onset. PLoS One.7,e43830. |
|  |  | Li,A., Krishna,A.D., Jun S.,et al.(2012).Pilot results of in vivo brain glutathione measurements in stroke patients. Journal of Cerebral Blood Flow & Metabolism.32,2118-2121. |
| Histidine metabolism | His metab | Naoto,A., Keyue,L., Kanji,N.,et al.(2011).Reduction of the infarct size by simultaneous administration of L-histidine and diphenhydramine in ischaemic rat brains. Resuscitation.2,219–221. |
|  |  | Naoto,A., Keyue,L., Tatsuru,A.,et al. (2005).Prevention of brain infarction by postischemic administration of histidine in rats. Brain Research.1–2, 220–223. |
|  |  | Naoto,A., Keyue,L.,Tatsuru,A.,et al.(2004).Alleviation of ischemic neuronal damage by postischemic loading with histidine in the rat striatum. Brain Research.1, 136–138. |
| Citrate cycle | TCA cycle | Mingxing,Y.,Shu,W.,Fuhua,H.,et al.(2012).NMR analysis of the rat neurochemical changes induced by middle cerebral artery occlusion. Talanta.,136–144. |
| Arachidonic acid metabolism | ArA metab | Gaetani,P.,Marzatico,F.,Rodriguez,R.,Pacchiarini,L.,Viganò,T.,et al.(1990).Arachidonic acid metabolism and pathophysiologic aspects of subarachnoid hemorrhage in rats.Stroke.21,328-332 |
|  |  | Earl,F.E., Kathy,F.W., Enoch,P.W.,Hermes,A. K.(1981).Cyclooxygenase Products of Arachidonic Acid Metabolism in Cat Cerebral Cortex After Experimental Concussive Brain Injury.Journal of Neurochemistry.37, 892–896. |
| Cysteine and methionine metabolism | CyM metab | Nida T., Sureyya E., Esra A.,et al.(2009).Hyperhomocysteinemia as an independent risk factor for cardioembolic stroke in the Turkish population. The Tohoku Journal of Experimental Medicine.4, 293-300. |
|  |  | LENTZ,S.R. (2005).Mechanisms of homocysteine-induced atherothrombosis. Journal of Thrombosis and Haemostasis.8,1646–1654. |
|  |  | Beccia,M.,Mele,M. C.,Ferrari,M., et al.(2004).Young stroke and basal plasma and post-methionine load homocysteine and cysteine levels 1 year after the acute event: do plasma folates make the difference? European Journal of Neurology.4,69–275. |
| Pyruvate metabolism | Pyr metab | Ruy,J., Cruz,Jr.,Tomoyuki,H.,Eizaburo,S.,et al.(2011). Effects of ethyl pyruvate and other α-keto carboxylic acid derivatives in a rat model of multivisceral ischemia and reperfusion. Journal of Surgical Research.1, 151–157. |
| Linoleic acid metabolism | LiA metab | Goede,J.D.,Verschuren,W.M.,Boer,J.M.,et al.(2013).N-6 and n-3 fatty acid cholesteryl esters in relation to incident stroke in a Dutch adult population: A nested case-control study. Nutrition, Metabolism and Cardiovascular Diseases.8,737–743. |
|  |  | Waylon,T., Hunt,A.K., Hope,D.A.,et al.(2010).Protection of cortical neurons from excitotoxicity by conjugated linoleic acid. Journal of Neurochemistry.1,123–130. |

Fig. S1 the flowchart of system-wide assembly of pathways and modules for Cerebral Ischemia


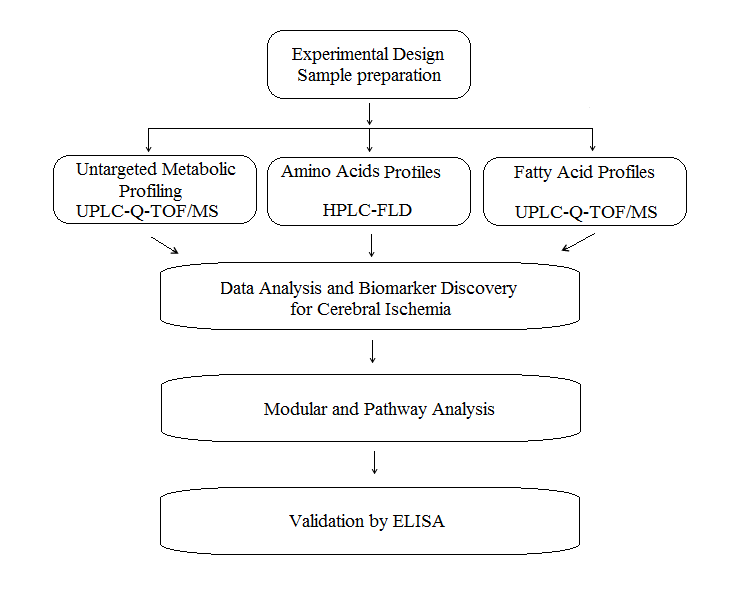


Fig. S2 The stroke shape and size stained with TTC
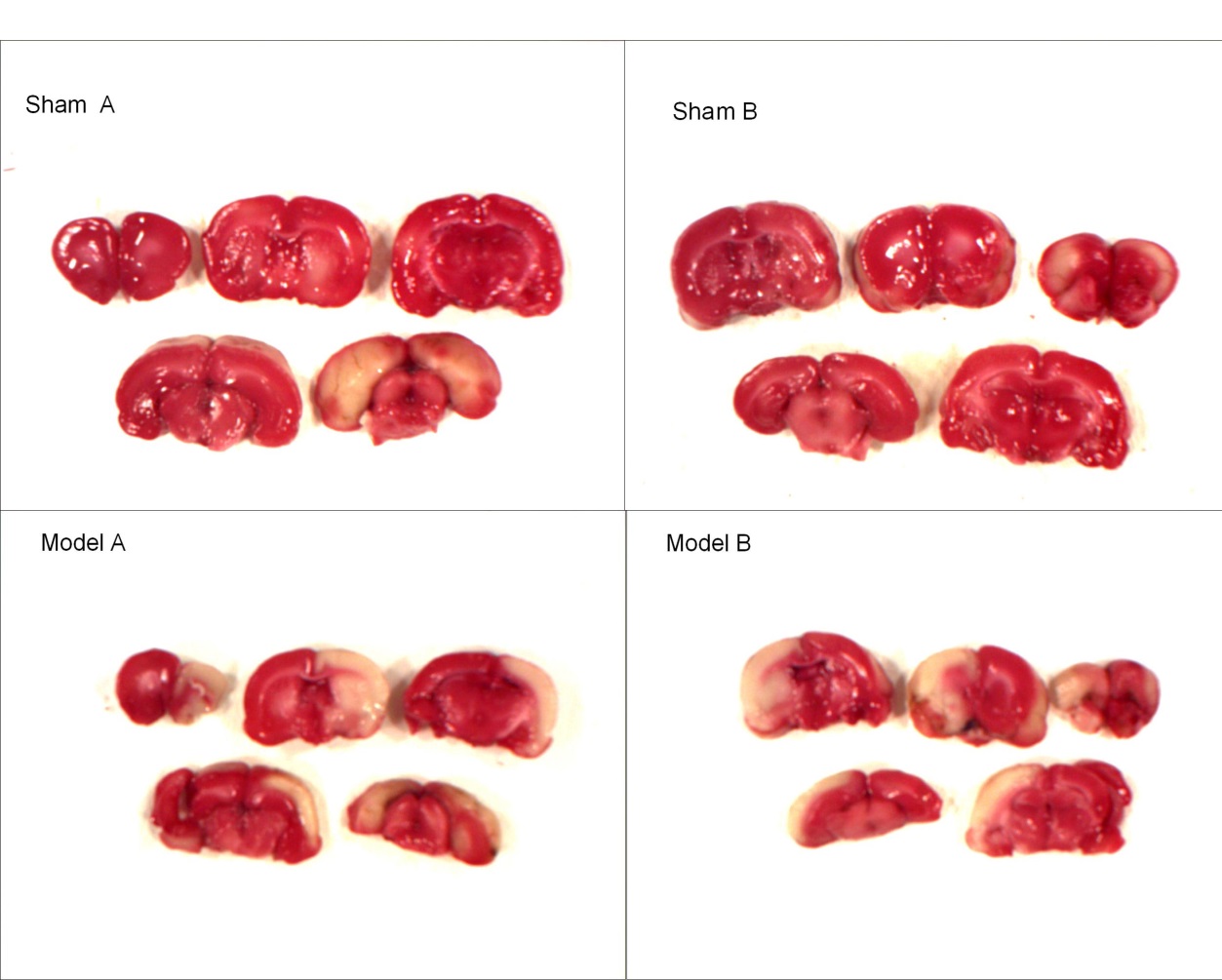


Fig. S3 Loading plot of serum fatty acid variables from PLS models of the sham and model groups. In this figure, variables far from 0.0 are more important than others.


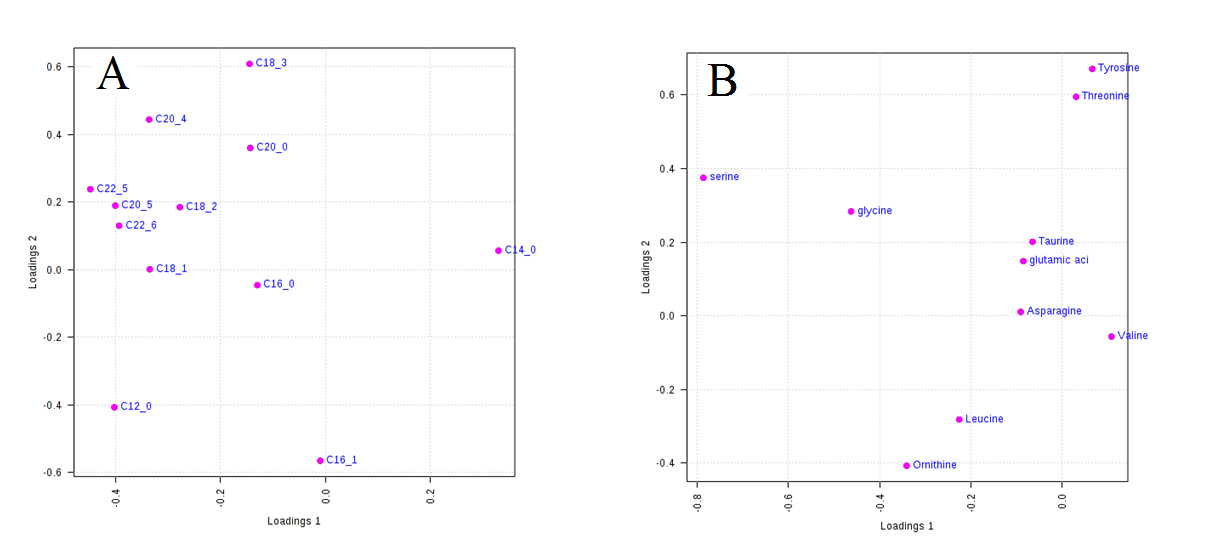


Fig. S4 Metabolic ischemic pathways analyzed with MetPA

**
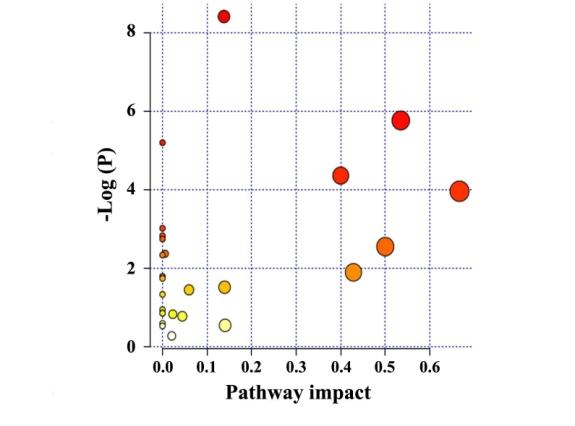
**

Fig. S5 Compound network map output by the Metscape under the operation of Cytoscape. Blue hexagon is a seed. Blue hexagon is a seed which means the variables selected as the potential biomarker. Pink hexagon is the compound related to the blue ones, which means there are some reactions between them.


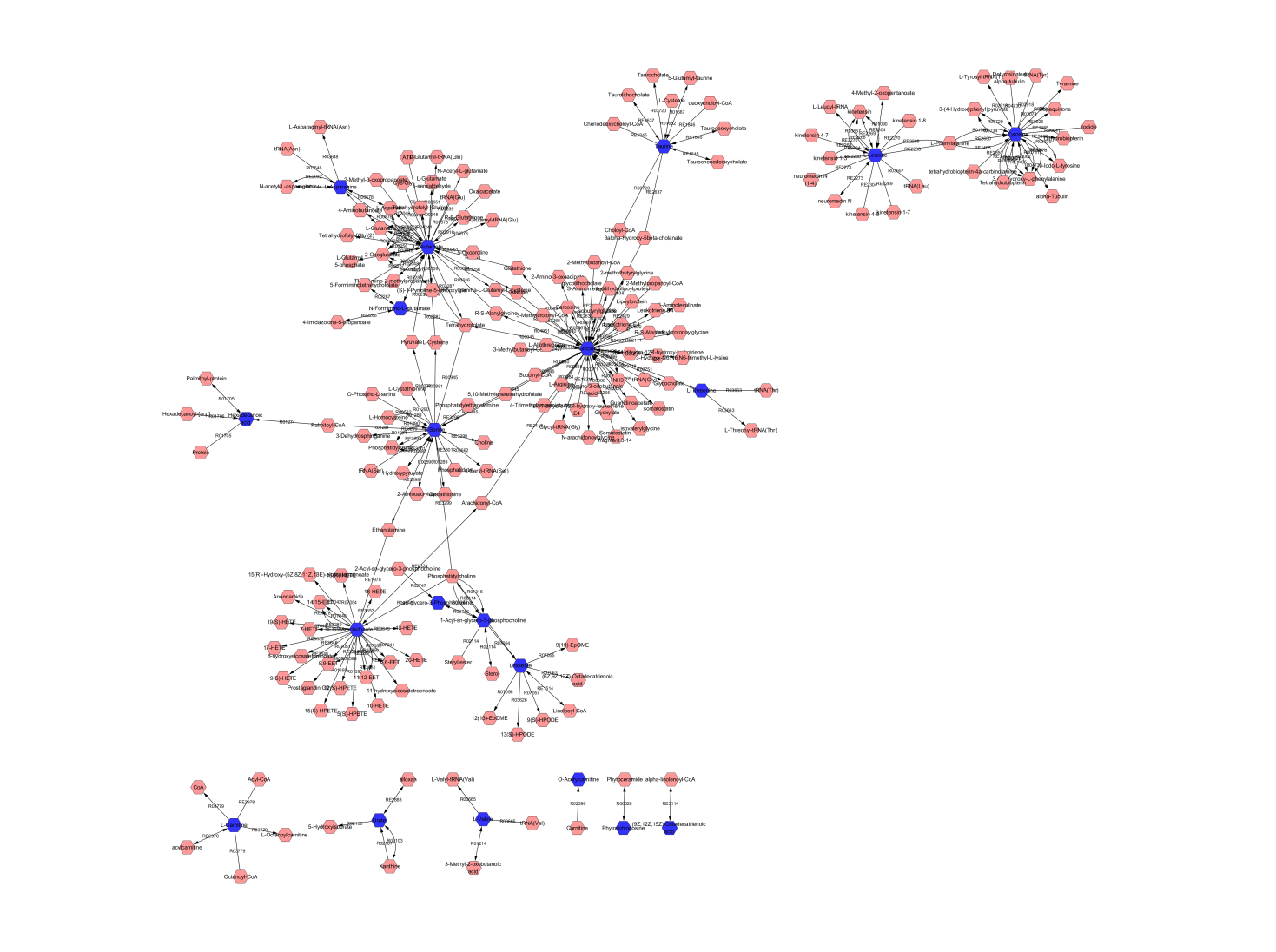


Fig. S6 Modular pattern of ischemic stroke by MCL. Totally 15 modules are identified from MCL method, and we give the names for the modules by the center compound in the module, such as glycine module, glutamate module, arachidonate module, serine module, tyrosine module, leucine module, taurine module, linoleate module, carnitine module, hexadecanoic acid module, asparagine module, valine module, urate module, threonine module and phosphocholine module.


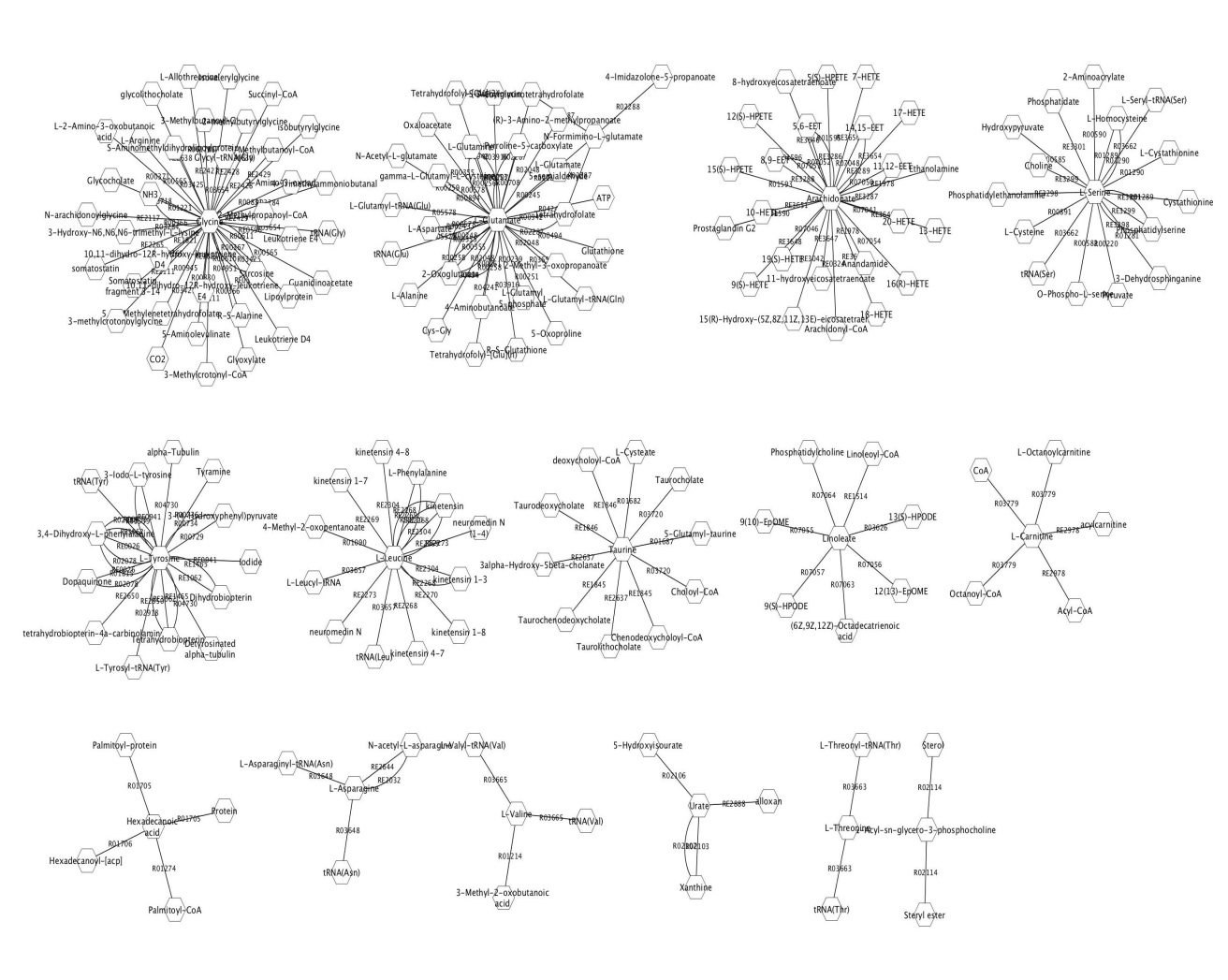


Fig. S7 (A) Comparing the nodes similarity between the glycine module and the glycine, serine, alanine, threonine metabolism pathways in Cytoscape. There are 22 identical nodes (47%) between the glycine module and GSAT MP, and the similarity is 63.9% computed by vectorial angle method.

Fig. S7 (B) Comparing the nodes similarity between the glutamate module and the urea cycle and metabolism of arginine, proline, glutamate, aspartate and asparagine pathways in Cytoscape. There are 20 identical nodes (51%) between the glutamate module and TCA&APGAA path, and the similarity is 67.8% computed by vectorial angle method.


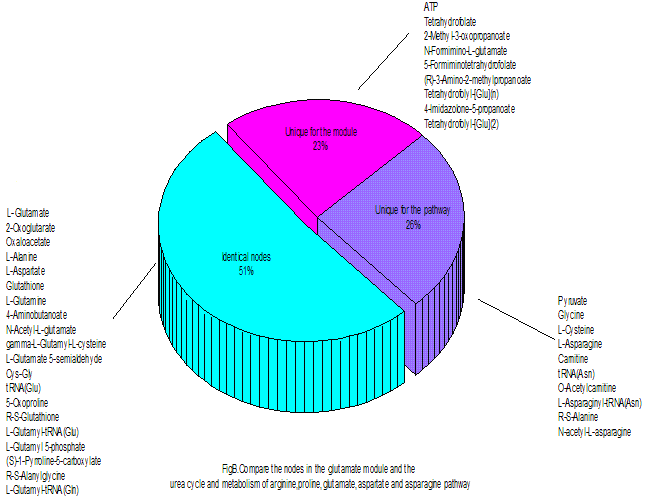


Fig. S7 (C) Comparing the nodes similarity between the arachidonate module and the arachidonic acid pathways in Cytoscape. There are 19 identical nodes (76%) between the arachidonate module and ArA path, and the similarity is 86.7% computed by vectorial angle method.

Fig. S7 (D) Comparing the nodes similarity between the serine module and the urea cycle and the metabolism of arginine, proline, glutamate, aspartate and asparagine pathways in Cytoscape. There are 2 identical nodes (5%) between the serine module and TCA&APGAA path, and the similarity is 9.1% computed by vectorial angle method.


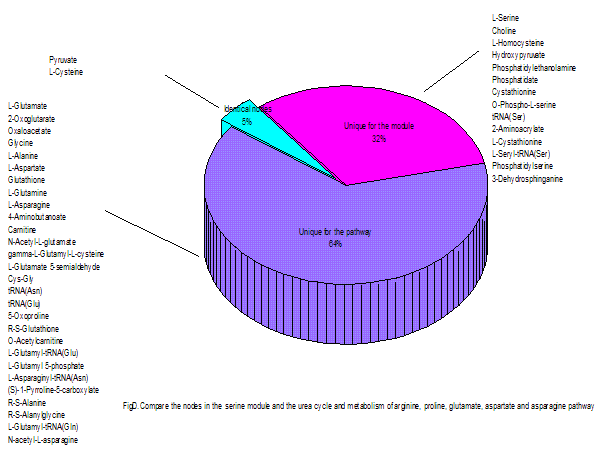


Fig. S7 (E) The tyrosine module has 11 overlapping nodes with the tyrosine metabolism pathway in Cytoscape. There are 11 identical nodes (73%) between the tyrosine module and Tyr MP_(metscape)_, and the similarity is 84.9% computed by vectorial angle method

Fig. S7 (F) Comparing the nodes similarity between the leucine module and the valine, leucine and isoleucine metabolism pathways in Cytoscape. There are 4 identical nodes (24%) between the leucine module and VLI MP_(metscape)_, and the similarity is 39.2% computed by vectorial angle method


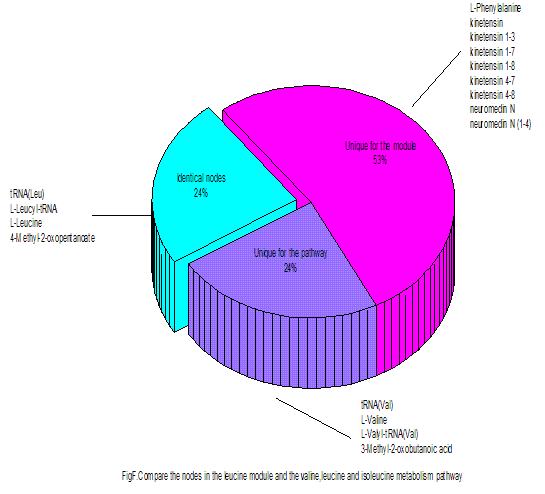


Fig. S7 (G) Comparing the nodes similarity between the taurine module and the bile acid biosynthesis pathway in Cytoscape. There are 9 identical nodes (64%) between the taurine module and BA biosyn, and the similarity is 78.3% computed by vectorial angle method.

Fig. S7 (H) Comparing the nodes similarity between the linoleate module and the linoleate metabolism pathway in Cytoscape. There are 7 identical nodes (78%) between the linoleate module and Lin MP, and the similarity is 87.5% computed by vectorial angle method
